# A chemoenzymatic synthesis of ceramide trafficking inhibitor HPA-12

**DOI:** 10.3762/bjoc.15.42

**Published:** 2019-02-18

**Authors:** Seema V Kanojia, Sucheta Chatterjee, Subrata Chattopadhyay, Dibakar Goswami

**Affiliations:** 1 Bio-Organic Division, Bhabha Atomic Research Centre, Mumbai 400 085, India; 2 Homi Bhabha National Institute, Training School Complex, Anushakti Nagar, Mumbai 400 094, India

**Keywords:** AD mix-β, [bmim][PF_6_], DDQ, HPA-12, lipase

## Abstract

A chemoenzymatic synthesis of the title compound has been developed using an efficient and highly enantioselective lipase-catalyzed acylation in a hydrophobic ionic liquid, [bmim][PF_6_], followed by a diastereoselective asymmetric dihydroxylation as the key steps for incorporating the stereogenic centers. The further conversion to the appropriate intermediates and subsequent acylation with lauric acid furnished the target compound.

## Introduction

**Figure 1:**
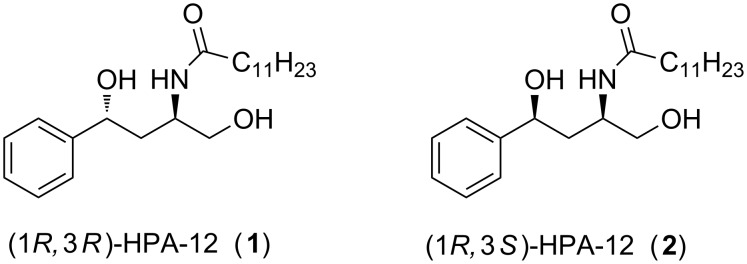
Structure of most active HPA-12 isomers, originally proposed (**1**) and revised (**2**).

Ceramides belong to the family of sphingolipids (SLs) and are synthesized de-novo in the endoplasmic reticulum (ER) [1]. Once formed, ceramide transport protein (CERT), a 68 kDa cytosolic protein, delivers the compound to the Golgi apparatus for further conversion to sphingomyelins, which play important roles in cell-signaling pathways [2]. In the last few years, the downregulation of CERT-mediated ceramide transfer from the ER to Golgi has gained increased attention in antioncogenic as well as antineurodegenerative therapeutic research [3–10]. However, chemical entities which inhibit CERT are scarce. In 2001, Hanada et al. reported (1*R*,3*R*)-*N*-(3-hydroxy-1-hydroxymethyl-3-phenylpropyl)dodecanamide (HPA-12, **1**, Figure 1) as the first inhibitor of CERT-mediated ceramide transport [11]. However, the initially determined (1*R*,3*R*) configuration of the most active HPA-12 stereoisomer (compound **1**, Figure 1) was later revised to (1*R*,3*S*) configuration (compound **2**, Figure 1) by Berkeš et al. in 2011 [12]. Since then, HPA-12 has been the subject of extensive biological evaluation. The HPA-12-mediated CERT knockdown has been associated with restoration of cell death in paclitaxel-resistant ovarian cancer cells [3] and also with the increased rate of ceramide-induced apoptosis following UVB irradiation, suggesting the possibility of its use as an anticancer compound. Besides, inhibition of sphingolipid biosynthesis using HPA-12 has also been reported to inhibit hepatitis C virus replication substantially [4]. In another study, the CERT knockdown disrupted the normal oxidative stress response in Drosophila [5]. More recently, a fluorinated analogue of HPA-12 has been studied for its BBB permeability and subsequent brain uptake, showcasing its possible use in neurodegenerative disorders [9].

However, a limited commercial availability and high cost of HPA-12 have hindered its application in basic researches involving CERT inhibition. For this, a number of groups have successfully accomplished the synthesis of HPA-12 [13–25]. The first synthesis of HPA-12 comprised [13] a three-component asymmetric Mannich reaction catalyzed by a chiral zirconium catalyst. However, after the structural revision of the most active stereoisomer, Kobayashi et al. synthesized (1*R*,3*S*)-HPA-12 (**2**, Figure 1) using a Zn-catalyzed asymmetric Mannich-type reaction in water, and unambiguously ascertained the revised configuration by X-ray crystallography [14]. The other syntheses of (1*R*,3*S*)-HPA-12 (**2**) used the chiral pool approach [15–16], crystallization-induced asymmetric transformation [17], diastereoselective reduction of γ-aryl-γ-oxo-β-amino alcohol [18], cycloaddition of oxime with alkenes [19], enantioselective carbonyl reduction followed by an organocatalyzed α-amination reaction [20], tandem approach from (*S*)-Wynberg lactone [21], chiral ruthenium-catalyzed *N*-demethylative rearrangement of 1,2-isoxazolidines [22], gold(I)-catalyzed cyclization of a propargylic *N*-hydroxylamine [23], from β-sulfinamido ketones derived from chiral sulfinimines [24], and a Kornblum–DeLaMare/aza-Michael reaction of 3,6-dihydro-1,2-dioxines followed by diastereoselective reduction [25]. Most of these methods employ starting materials or catalysts, which are not commercially available, and also require operationally demanding reaction conditions. Hence a need to develop a simple, efficient and inexpensive synthesis of HPA-12 was felt. Our own interest in developing anticancer agents also prompted us to develop a new and practical enantioselective synthesis of **2** [26–29].

To realize our objective, we paid particular attention to obtain **2** using reactions that are high-yielding and can be executed under simple reaction conditions with commercially available and inexpensive materials/reagents. In this regard biocatalytic reactions offer green and sustainable alternative routes to develop asymmetric syntheses of pharmaceuticals with varied stereochemical features [30–33]. Our group has been using lipases for the chemoenzymatic syntheses of several bioactive molecules [34–39]. The prevalence of PhCH(OH) in **2**, and in many other biochemicals, attracted our attention to formulate an enantioselective lipase-catalyzed transacetylation strategy to obtain a suitable molecule bearing the designated chiral segment (compound **2**, vide infra).

## Results and Discussion

**Scheme 1:**
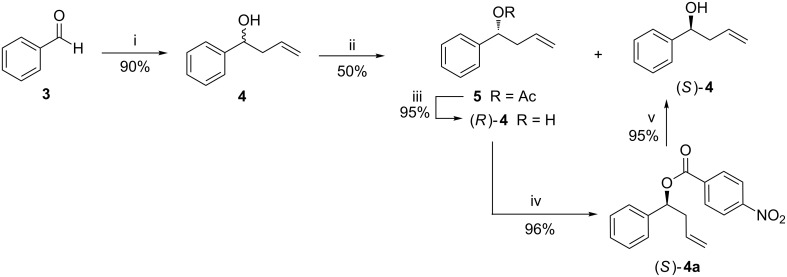
Lipase-catalyzed trans-acylation of (±)-**4** and subsequent Mitsunobu inversion. Conditions: (i) Zn/THF/allyl bromide/aqueous sat. NH_4_Cl/25 °C/3 h; (ii) vinyl acetate/lipase (Table 1); (iii) KOH/EtOH/25 °C/6 h; (iv) Ph_3_P/DIAD/*p*-nitrobenzoic acid/THF; (v) KOH/EtOH/25 °C/8 h.

For the synthesis, commercially available benzaldehyde (**3**) was allylated using Zn/allyl bromide in moist THF following Luche’s protocol to obtain the homoallylic alcohol (±)-**4** (Scheme 1) [40]. We envisaged that the enantiomers of **4** were ideal substrates for the asymmetric synthesis of different stereoisomers of **2**. Several biocatalytic protocols for the preparation of (*R*) or (*S*)-**4** were reported earlier. The hydrolysis of the corresponding acetate with crude enzyme preparations from pig liver and chicken liver esterase proceeded with modest enantioselectivity [41–42]. *Rhizopus arrhizus*-mediated hydrolysis of the acetate furnished the enantiomerically pure alcohol (99% ee), however, the enantiomeric excess (ee) of the antipode acetate was very poor (5–9%) [43]. On the other hand, the Amano PS lipase-catalyzed trans-acylation of (±)-**4** furnished (*S*)-**4** with good ees, while the ee of the (*R*)-acetate was poor [44]. The acylase 1-catalyzed resolution protocol gave (*S*)-**4** in a poor % ee [45]. A unified approach where both the enantiomers of **4** are obtained in good optical purities is rare. This is important as the individual enantiomers can be converted to the antipodes by Mitsonobu inversion [46], thereby maximizing the yield of the desired enantiomer. In addition, the availability of both the enantiomers of **4** would be useful for the synthesis of all diastereomers of **2** (as per our synthetic plan) and also several other pharmacologically important compounds. To the best of our knowledge, only the lipase PS-catalyzed acylation of (±)-**4** proceeded with good to excellent ees for both the acetate and alcohol. However, details of the protocol are unavailable, and also the resolution was quite slow [47]. Hence, we screened different lipases for the trans-acetylation of (±)-**4** for its effective resolution and the results are summarized in Table 1. Based on our past experience in lipase-catalyzed resolution of homoallylic alcohols [38], initially a Novozym 435^®^-catalyzed acetylation of (±)-**4** with vinyl acetate in diisopropyl ether (DIP) was attempted. However, the yield and enantioselectivity of the desired alcohol (*S*)-**4** were very poor (Table 1, entry 1). Also the acetylation of (±)-**4** with vinyl acetate in diisopropyl ether using *Candida rugosa* lipase (CRL) and *Pseudomonas fluorescens* lipase (PFL) were futile (Table 1, entries 2 and 3). In contrast, a better yield and enantiocontrol was achieved using Amano lipase from *P. fluorescens* (Amano PFL) in conjunction with vinyl acetate in diisopropyl ether, where the (*R*)-acetate **5** and (*S*)-alcohol **4** were obtained in 85% and 88% ee, respectively, at 48% conversion after 192 h (Table 1, entry 4). The efficacy of Amano PFL for the resolution of (±)-**4** is in corroboration with the reported method using Amano lipase PS [44]. However, since the reaction was very slow, we attempted to improve the reaction rate by carrying out the resolution at 50 °C, near the optimum temperature of the enzyme (55 °C). Under these conditions, a 50% conversion was achieved within 48 h to obtain both (*R*)-**5** and (*S*)-**4** in 90% ee (Table 1, entry 5). A second acetylation (12% conversion, 20 h) of the resolved (*S*)-**4** under the same conditions improved its ee to 99% (Table 1, entry 6). The products were isolated by filtering the insoluble enzyme from the reaction mixture, followed by concentration of the filtrate. The obtained residue was subjected to silica gel column chromatography to isolate unreacted alcohol and acetylated product. We have reused the recovered lipase at least three times without any significant loss of enzyme activity.

**Table 1:** Lipase-catalyzed resolution of (±)-**4**.

Entry	Lipase	Acylating agent	Solvent	Time (h)	% Conversion^a^	% ee of (*S*)-**4**^a^	% ee of (*R*)-**5**^a^	% c^b^	Yield of enriched (*S*)-**4**^c^	Yield of enriched (*R*)-**5**^c^	E^d^

1	Novozym 435	vinyl acetate	DIP	96	20	22	71	23.7	76	16	7.21
2	CRL	vinyl acetate	DIP	72	23	19	68	21.8	71	20	6.35
3	Lipase from *P. fluorescens*	vinyl acetate	DIP	96	26	35	89	28.2	67	21	24.58
4	Amano PFL	vinyl acetate	DIP	192^e^	48	88	85	50.9	45	42	35.40
5	Amano PFL	vinyl acetate	DIP	48^f^	50	90	90	50.0	46	43	58.75
6	Amano PFL	vinyl acetate	DIP	20	12^g^	99	ND	ND	82	10	ND
7	Amano PFL	vinyl acetate	[bmim] [BF_4_]	48	10^e^	ND	ND	ND	ND	ND	ND
8	Amano PFL	vinyl acetate	[bmim] [PF_6_]	6^f^	48	92	91	50.3	45	42	68.62

^a^Experimentally determined from chiral HPLC analysis using AD-H column and 5% isopropanol/hexane as eluent @1.0 mL min^−1^, λ = 254 nm. ^b^The % c values were calculated from the enantiomeric excess of the starting material (ee_s_) and the product (ee_p_) according to % c = ee_s_/(ee_s_ + ee_p_). ^c^Isolated yield. ^d^The enantiomeric factor (E) was calculated from the enantiomeric excess of the starting material (ee_s_) and the c value according to E = ln[(1 − c)(1 − ee_s_)]/ln[(1 − c)(1 + ee_s_). ^e^Reaction done at 25 °C. ^f^Reaction done at 50 °C. ^g^Reaction done on the partially resolved alcohol obtained from the previous entry.

There are similar instances in literature [48] for a substantial slow-down of the transesterification reaction rate in organic solvents. This issue can be overcome by using room temperature ionic liquids, which not only substitute the environment damaging organic solvent, but also increase the reaction rate, and provide many other technological advantages [49]. Towards this, we have chosen [bmim][BF_4_] and [bmim][PF_6_] as two model ionic liquids. Of them, [bmim][BF_4_] is water soluble, and [bmim][PF_6_] is immiscible with water. The acetylation of (±)-**4** with vinyl acetate in [bmim][BF_4_] using Amano PFL was very slow (Table 1, entry 7), showing only 10% completion after 48 h. Carrying out the reaction at 50 °C also did not improve the reaction rate. However, the acetylation in [bmim][PF_6_] at 50 °C using vinyl acetate and Amano PFL was much faster (Table 1, entry 8), and a 48% conversion was achieved in only 6 h to obtain (*R*)-**5** and (*S*)-**4** in 91% and 92% ee, respectively. This kind of dependency of the reaction rate on the nature of the anion in the ionic liquid has been reported earlier [50]. Whatsoever, the reaction protocol in [bmim][PF_6_] was fruitful for the successful resolution of racemic (±)-**4**. The products were extracted from the [bmim][PF_6_] medium with diethyl ether and the ethereal phase was concentrated. The residue was then subjected to silica gel column chromatography to obtain the unreacted alcohol and the acetate. We have reused the recovered lipase in ionic liquid at least three times without any significant loss of enzyme activity.

The % ee of the (*R*)-**5** and (*S*)-**4** were determined from chiral HPLC analyses (150 mm × 4.6 mm, 5 μm, chiral AD-H column, 5% isopropanol/hexane @ 1.0 mL min^−1^, UV detection at 254 nm). The absolute configurations of (*R*)-**5** and (*S*)-**4** were assigned by comparison of the chiroptical data with those reported [45]. The stereochemical outcome of the reaction is consistent with Kazlauskas’ empirical rule [51]. The conversion (% c) and the enantiomeric excess (E) values were calculated according to the method described by Sih et al. [52]. Besides the high yield and % ee, the biocatalytic protocol is operationally simple and convenient. We carried out the transformation using inexpensive vinyl acetate as the acyl donor due to its volatility that would assist easy isolation of the products. All the reactions were carried out at least 3–4 times and the best results are presented in Table 1. To the best of our knowledge, this is the first attempt towards the resolution of (±)-**4** in a room temperature ionic liquid at an elevated temperature.

**Scheme 2:**
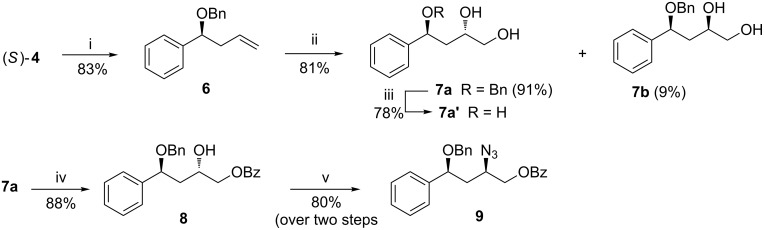
Synthesis of azide **9** from (*S*)-**4**. Conditions: (i) NaH/Bu_4_NI/BnBr/THF/25 °C/4 h; (ii) AD-mix-β/*t*-BuOH–H_2_O 1:1/0 °C/72 h; (iii) DDQ/CH_2_Cl_2_–H_2_O 4:1/3 h; (iv) Et_3_N/BzCN/0 °C/CH_2_Cl_2_/2 h; (v) a) MsCl/Et_3_N/CH_2_Cl_2_/0 °C/2.5 h, b) NaN_3_/DMF/90 °C/3 h.

To make the synthesis enantio-convergent, and also to offset the limitations of a resolution-based protocol, (*R*)-**5** was hydrolyzed with alcoholic KOH to furnish (*R*)-**4** (Scheme 1). Its inversion under the Mitsunobu conditions (Ph_3_P/DIAD/*p*-nitrobenzoic acid/THF; KOH/EtOH/25 °C/8 h, 91% over two steps) gave (*S*)-**4** [46]. The benzylation of the hydroxy function in (*S*)-**4** with benzyl bromide (BnBr) and Bu_4_NI in the presence of NaH produced compound **6** (Scheme 2). This was subjected to asymmetric dihydroxylation (ADH) using AD mix-β [K_2_OsO_2_(OH)_4_ and (DHQD)_2_-PHAL]. The reaction proceeded predominantly from the α-face, resulting in the formation of the 1,3-*anti* diol **7a** and 1,3-*syn* diol **7b** in a 91:9 ratio (based on the isolated yields of **7a** and **7b**, separated by column chromatography). Previously the ADH reaction of a homologue of **6**, bearing a methyl substitution and a hydroxy group (instead of benzyloxy group) with AD mix-β also produced the corresponding α-alcohol [53]. However, unlike in our case, the reaction proceeded with poor diastereoselectivity irrespective of the dihydroxylating agent used. To confirm the 1,3-*anti* diol stereochemistry of **7a**, it was debenzylated using DDQ/CH_2_Cl_2_–H_2_O to furnish the trihydroxy compound **7a'**. The ^1^H and ^13^C NMR spectra, and the optical rotation of **7a'** were in conformity with those reported [54]. In addition, the 1,3-*anti* stereochemistry of the diol **7a** was confirmed by converting it to the target compound **2**, and comparing its chiroptical data with the reported values, as described afterwards.

**Scheme 3:**
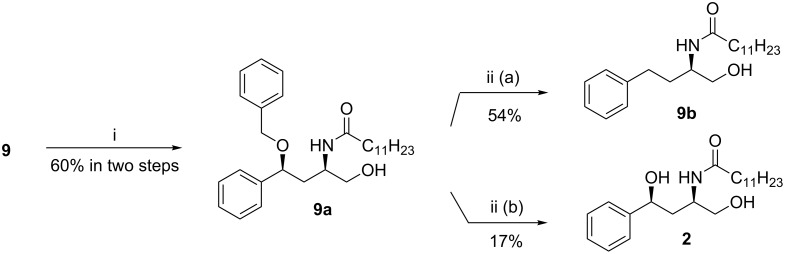
Attempted synthesis of **2** from **9**. Conditions: (i) (a) LiAlH_4_ (1 M in THF)/THF/25 °C/3 h, (b) DCC/DMAP/lauric acid, CH_2_Cl_2_/25 °C/18 h; (ii) (a) H_2_/10% Pd–C/EtOH/25 °C or (b) DDQ/CH_2_Cl_2_–H_2_O 4:1/3 h.

**Scheme 4:**
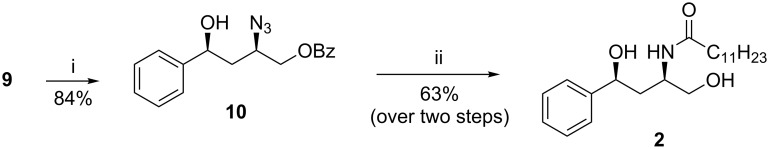
Actual synthesis of **2** from **9**. Conditions: (i) DDQ/CH_2_Cl_2_–H_2_O 4:1/3 h; (ii) a) LiAlH_4_/THF/25 °C/3 h, b) DCC/DMAP/lauric acid, CH_2_Cl_2_/25 °C/18 h.

For the synthesis of **2**, the primary hydroxy function of **7a** was benzoylated to get **8**. Compound **8** was mesylated with methanesulfonyl chloride (MsCl)/Et_3_N and the product reacted with NaN_3_/DMF at 90 °C to obtain the azide **9** (Scheme 2). We first attempted to convert **9** to the target compound **2** by i) converting the azide group to the amine using LiAlH_4_ with concomitant debenzoylation, followed by the acylation of the amine with lauric acid to afford **9a**, and finally, ii) reductive cleavage by hydrogenolysis using Pd–C/H_2_ leading to debenzylation (Scheme 3). However, during hydrogenolysis, the elimination of the -OBn group led to product **9b**, which was undesirable. A similar elimination was earlier observed by Sharf et al. during hydrogenolysis of dibenzyl ether [55]. To avoid this, an oxidative debenzylation of **9a** using DDQ/CH_2_Cl_2_–H_2_O was carried out. However, this led to a very poor yield of the target compound **2** (Scheme 3). Hence, we decided to debenzylate compound **9** prior to its reduction to the amine and subsequent acylation. Towards this (Scheme 4), oxidative debenzylation of **9** using DDQ yielded **10** in 84% yield. Treatment of **10** with LiAlH_4_ led to the reduction of the azide group to amine along with debenzoylation to furnish an intermediate aminodiol, which, without further purification, was acylated with lauric acid in the presence of dicyclohexylcarbodiimde (DCC) and 4-dimethylaminopyridine (DMAP) to give the target compound HPA-12 (1*R*,3*S*)-**2**. The optical and spectroscopic data of compound **2** were in accordance with those reported [18].

## Conclusion

In summary, we have demonstrated an efficient protocol for the synthesis of HPA-12 using a lipase-catalyzed resolution of the alcohol (±)-**4** in an ionic liquid and a diastereoselective ADH reaction as the key steps. The synthesis was accomplished by employing reactions that use inexpensive reagents, are operationally simple and proceed with good to excellent yields and excellent stereoselectivities. The target compound was obtained in 23% overall yield starting from (*S*)-**4**. The protocol can also be used to access different HPA analogues and derivatives.

## Supporting Information

File 1 Experimental details and analytical data.

File 2 NMR spectra.
