# Supplementary material for: A chemoenzymatic synthesis of ceramide trafficking inhibitor HPA-12
Source: Beilstein J Org Chem. 2019 Feb 18;15:490–6. doi: 10.3762/bjoc.15.42 (PMC6404422; doi:10.3762/bjoc.15.42)
Supplement: File 1 — Experimental details and analytical data. [file Beilstein_J_Org_Chem-15-490-s001.pdf]

BEILSTEIN JOURNAL OF ORGANIC CHEMISTRY

Supporting Information

for

A chemoenzymatic synthesis of ceramide trafficking inhibitor HPA-12

Seema V. Kanojia, Sucheta Chatterjee, Subrata Chattopadhyay and Dibakar Goswami

Beilstein J. Org. Chem. **2019,** *15,* 490–496. doi:10.3762/bjoc.15.42

Experimental details and analytical data

License and Terms: This is a supporting information file under the terms of the Creative Commons Attribution License (http://creativecommons.org/licenses/by/4.0). Please note that the reuse, redistribution and reproduction in particular requires that the authors and source are credited.
The license is subject to the *Beilstein Journal of Organic Chemistry* terms and conditions: (https://www.beilstein-journals.org/bjoc)

Experimental section

All chemicals were procured from Sigma Aldrich and were used as received. Other reagents were of AR grade. All anhydrous reactions were carried out under an Ar atmosphere using freshly dried solvents. All organic extracts were dried over anhydrous Na_2SO_4. IR spectra were recorded as films with a BRUKER Tensor II spectrophotometer. ^1H and ^{13}C NMR spectra were recorded in $CDCl_3$ with either a Varian 500 MHz or a Bruker AC-200 instrument, and were processed using the Bruker TOPSPIN software. Optical rotations were recorded on a JASCO DIP-360 digital polarimeter. Elemental analyses were recorded on an Elementar Vario microcube. The chiral HPLC analyses were performed with a JASCO HPLC system using a CHIRALCEL AD-H column with HPLC grade *n*-hexane and isopropanol as the eluents.

1-Phenylbut-3-en-1-ol (4).[1] To a stirred solution of benzaldehyde (**3**, 4.00 g, 37.72 mmol) and allyl bromide (8.15 mL, 94.29 mmol) in THF (50 mL), Zn powder (4.93 g, 75.44 mmol) was added portionwise. The suspension was cooled (10 °C) and a few drops of aqueous saturated NH_4Cl were added. The reaction mixture was brought to room temperature (25 °C) and magnetically stirred for additional 3 h. After completion of the reaction (cf. TLC, 10% EtOAc/hexane), the reaction mixture was filtered, extracted with EtOAc (3 × 10 mL), the organic extract washed with H_2O (1 × 10 mL) and brine (1 × 5 mL), dried and concentrated in vacuo. The residue, obtained after concentration, was purified by column chromatography (silica gel, 0–10% EtOAc/hexane) to afford **4** as an inseparable enantiomeric mixture. Yield: 5.03 g (90%) colorless oil; IR (film): ν_{max} 3416, 3077, 3030, 1641 cm^{-1} ; ^1H NMR (200 MHz): δ 2.19 (broad s, 1H), 2.51-2.58 (m,2H), 4.74-4.80 (m, 1H), 5.15-5.23 (m, 2H), 5.78-5.91 (m, 1H), 7.29-7.40 (m, 5H); ^{13}C NMR (50 MHz): δ 43.8, 73.4, 118.3, 125.9, 127.6, 128.4, 134.5, 143.9.

General method for lipase-catalyzed acylation in organic media. A mixture of (±)-**4** (500 mg, 3.37 mmol), lipase (200 mg, specified in Table 1) and vinyl acetate (580 mg, 6.74 mmol) in diisopropyl ether (5 mL) was agitated on an orbital shaker at 110 rpm at the temperature and for the time period specified in Table 1. Then, the reaction mixture was filtered and the solution concentrated in vacuo to get a residue, which after column chromatography (silica gel, 0–15% Et_2O/hexane) gave pure (*S*)-**4** and (*R*)-**5**. Yields and % conversions are specified in Table 1. For re-resolution of (*S*)-**4** (entry 6, Table 1), the same conditions were maintained. Several batches of (*S*)-**4** were prepared using the resolution protocol with Amano lipase from Pseudomonas fluorescens at 50 °C, and the batches were pooled together for further steps.

General method for Amano lipase-catalyzed acylation in ionic liquid. A mixture of (±)-**4** (500 mg, 3.37 mmol), lipase (200 mg, specified in Table 1) and vinyl acetate (580 mg, 6.74 mmol) in either [bmim][BF_4] or [bmim][PF_6] (3 mL) was agitated on an orbital shaker at 110 rpm at the temperature and for the time period specified in Table 1. The products were extracted in Et_2O (3 × 5 mL) and the combined extract concentrated in vacuo to get a residue, which after column chromatography (silica gel, 0–15% Et_2O/hexane) gave pure (*S*)-**4** and (*R*)-**5**. Yields and % conversions are specified in Table 1.

Data for alcohol and acetate obtained from Amano lipase mediated resolution in [bmim][PF_6]:

(*S*)-1-Phenylbut-3-en-1-ol ((*S*)-**4**).[2] Colorless oil; $[\alpha]_D^{25}$ -28.8 (*c* 1.10, $CHCl_3$) [lit.[2] $[\alpha]_D^{25}$ -30.7 (*c* 0.533, $CHCl_3$)]; ee = 92% (determined by HPLC; column AD-H, eluent: isopropanol : hexane 5:95, flow rate 0.5 mL/min; (*R*)-enantiomer: t_R = 16.67 min, (*S*)-enantiomer: t_R = 17.12 min); IR (film): ν_{max} 3415, 1643 cm^{-1}; ^{1}H NMR (200 MHz): δ 2.32 (broad s,1H), 2.46-2.53 (m, 2H), 4.70 (t, *J* = 6.4 Hz, 1H), 5.10-5.18 (m, 2H), 5.69-5.90 (m, 1H), 7.30-7.35 (m, 5H); ^{13}C NMR (50 MHz): δ 43.3, 73.1, 117.5, 125.6, 127.1, 128.0, 134.3, 143.7. Anal. Calcd. for $C_{10}H_{12}O$: C, 81.04; H, 8.16%. Found: C, 81.03; H, 8.28%.

(*R*)-1-Phenyl-but-3-en-1-yl acetate ((*R*)-5).[2] Colorless oil; $[\alpha]_D^{27}$ +38.5 (*c* 1.20, $CHCl_3$) [lit.[2] $[\alpha]_D^{25}$ +41.6 (*c* 1.5, $CHCI_3$)]; ee = 91% (determined by HPLC; column AD-H, eluent: isopropanol : hexane 5:95, flow rate 0.5 mL/min; (*R*)-enantiomer: t_R = 8.21 min, (*S*)-enantiomer: t_R = 8.74 min); IR (film): ν_{max} 1732, 1643 cm^{-1}; ^{1}H NMR (200 MHz): δ 2.07 (s,3H), 2.51-2.73 (m, 2H), 5.02-5.11 (m, 2H), 5.60-5.84 (m, 2H), 7.25-7.35 (m, 5H); ^{13}C NMR (50 MHz): δ 20.6, 40.3, 74.6, 117.5, 126.1, 127.5, 128.0, 133.0, 139.7, 169.5. Anal. Calcd. for $C_{12}H_{14}O_2$: C, 75.76; H, 7.42%. Found: C, 75.48 ; H, 7.55 %.

(*R*)-1-Phenylbut-3-en-1-ol ((*R*)-**4**).[3] A solution of (*R*)-**5** (500 mg, 2.63 mmol) in aqueous ethanolic KOH (2 M, 5 mL) was stirred at room temperature for 6 h (reaction progress monitored using TLC, 10% EtOAc/hexane). Most of the solvent was removed in vacuo and the residue extracted into $CHCl_3$ (10 mL). The organic extract was washed with water (2 × 5 mL) and brine (1 × 5 mL), and dried. Removal of solvent followed by column chromatography of the residue (silica gel, 0–10% EtOAc/hexane) furnished pure (*R*)-**4**. Yield: 370 mg (95%); $[\alpha]_D^{25}$ +33.4 (*c* 1.10, $CHCl_3$) [lit.[3] $[\alpha]_D^{25}$ +34.90 (c = 0.18, $CHCl_3$)]; IR (film): ν_{max} 3416, 1641 cm^{-1}; ^{1}H NMR (200 MHz): δ 2.22 (broad s,1H), 2.50-2.58 (m, 2H), 4.76 (t, *J* = 6.4 Hz, 1H), 5.14-5.24 (m, 2H), 5.74-5.94 (m, 1H), 7.28-7.40 (m, 5H); ^{13}C NMR (50 MHz): δ 43.8, 73.4, 118.3, 125.9, 127.6, 128.4, 134.5, 144.1.

(*S*)-1-Phenylbut-3-enyl-4-nitrobenzoate ((*S*)-**4a**).[4] To a stirred solution of (*R*)-**4** (300 mg, 2.03 mmol) in dried THF (20 mL), PPh_3 (639 mg, 2.44 mmol) and *p*-nitrobenzoic acid (408 mg, 2.44 mmol) were added under nitrogen atmosphere. Next, the reaction mixture was cooled to 0 °C and diisopropylazodicarboxylate (DIAD, 493 mg, 2.44 mmol) was added dropwise. The reaction mixture was further stirred at room temperature for 18 h (reaction progress monitored using TLC, 10% EtOAc/hexane). The solvent was evaporated under reduced pressure and the crude was purified by column chromatography (silica gel, 0–5% EtOAc/hexane). Yield: 579 mg (96%); ^{1}H NMR (500 MHz): δ 2.70-2.76 (m, 1H), 2.81-2.87 (m, 1H), 5.08-5.17 (m, 2H), 5.73-5.82 (m, 1H), 6.08 (t, *J* = 6.5 Hz, 1H), 7.31-7.33 (m, 1H), 7.34-7.39 (m, 2H), 7.42-7.44 (m, 2H), 8.22-8.24 (m, 2H), 8.28-8.30 (m, 2H); ^{13}C NMR (125 MHz): δ 40.8, 76.9, 118.6, 123.5, 123.6, 126.5, 128.4, 128.6, 130.7, 132.9, 135.8, 139.4, 150.5, 163.9.

As described before, the obtained *p*-nitrobenzoate ester ((*S*)-**4a**, 550 mg, 1.85 mmol) was dissolved in aqueous ethanolic KOH (2 M, 5 mL) and the reaction mixture stirred for 8 h (reaction progress monitored by TLC, 10% EtOAc/hexane), and worked-up as before. The

crude was purified by column chromatography (silica gel, 0–10% EtOAc/hexane) giving pure (*S*)-**4**. Yield: 260 mg (95%). The chiroptical data was as described before.

(*S*)-(1-Benzyloxy-but-3-enyl)benzene (6).[5] A solution of (*S*)-**4** (1.80 g, 12.15 mmol) in THF (30 mL) was added to a stirred suspension of NaH (0.73 g, 50% suspension in oil, 30.38 mmol) in THF (20 mL) under Ar, and the mixture refluxed for 1 h. Then it was brought to room temperature and a mixture of Bu_4NI (10 mol %) and BnBr (2.49 g, 14.58 mmol) dissolved in THF (30 mL) was dropwise added. The mixture was refluxed till the reaction was complete (cf. TLC, 10% EtOAc/hexane, ≈ 4 h). Then the reaction mixture was brought to room temperature, quenched with ice-cold H_2O (20 mL), extracted with EtOAc (3 × 10 mL), the organic extract washed with water (3 × 10 mL) and brine (1 × 5 mL), and dried. Solvent removal under vacuo followed by column chromatography (silica gel, 0-10% EtOAc-hexane) of the residue furnished **6**. Yield: 2.4 g (83%); colorless oil; $[\alpha]_D^{25}$ -69.2 (*c* 1.24, $CHCl_3$) lit.[4] $[\alpha]_D^{23}$ -69.8 (*c* 1.01, $CHCl_3$)]; IR (film): ν_{max} 3064, 3029, 1641,914 cm^{-1}; ^1H NMR (500 MHz): δ 2.46-2.49 (m,1H), 2.63-2.69 (m, 1H), 4.30 (d, *J* = 12.0 Hz, 1H), 4.39 (t, *J* = 7.0 Hz, 1H), 4.49 (d, *J* = 12.0 Hz, 1H), 5.02-5.08 (m, 2H), 5.77-5.85 (m, 1H), 7.26-7.40 (m, 10H); ^{13}C NMR (125 MHz): δ 42.6, 70.4, 81.2, 116.8, 126.9, 127.4, 127.6, 127.7, 128.3, 128.4, 134.9, 138.5, 141.9. Anal. Calcd. for $C_{17}H_{18}O$: C, 85.67; H, 7.61%. Found: C, 85.59; H, 7.81%.

4-(Benzyloxy)-4-phenylbutane-1,2-diol (7). AD-mix β (11.75 g, 1.4 g/mmol) was added stepwise to a cooled (0 °C) and stirred solution of **6** (2.00 g, 8.39 mmol) in 50% aqueous *t*-BuOH (20 mL). The mixture was stirred at 0 °C till completion of the reaction (*cf*. TLC, 30% EtOAc/hexane, ≈ 48 h). Then aqueous saturated Na_2SO_3 was added to quench the reaction, and the mixture was extracted with $CHCl_3$ (3 × 10 mL). The organic extract was washed with water (3 × 10 mL) and brine (1 × 5 mL), dried and concentrated in vacuo. The residue was purified by column chromatography (silica gel, 0–30% EtOAc/hexane) to afford pure **7a** and **7b** in a 91:9 diastereomeric ratio and 81% combined yield. **(2*S*,4*S*)-4-(Benzyloxy)-4-phenylbutane-1,2-diol (7a, major)** Yield: 1.69 g (74%); colorless oil; $[\alpha]_D^{25}$ -56.1 (*c* 0.97, $CHCl_3$); IR (film): ν_{max} 3417, 3063, 3030, 1603, 914 cm^{-1}; ^1H NMR (500 MHz): δ 1.76-1.83 (m,2H), 1.97 (s, 1H), 2.95 (broad s, 1H), 3.35 and 3.37 (two d, *J* = 7.0 Hz both, 1H), 3.51 and 3.53 (two d, *J* = 3.5 and 3.0 Hz, 1H), 3.90-3.93 (m, 1H), 4.21 (d, *J* = 11.5 Hz, 1H), 4.42 (d, *J* = 12.0 Hz, 1H), 4.60 and 4.61 (two d, *J* = 3.0 and 3.5 Hz, 1H), 7.22-7.31 (m, 10H); ^{13}C NMR (125 MHz): δ 41.2, 66.7, 69.1, 70.8, 78.4, 126.5, 127.7, 127.8, 127.9, 128.5, 128.6, 137.9, 141.6. Anal. Calcd. for $C_{17}H_{20}O_3$: C, 74.97; H, 7.40%. Found: C, 74.94; H, 7.62%. **(2*R*,4*S*)-4-(Benzyloxy)-4-phenylbutane-1,2-diol (7b, minor):** Yield: 160 mg (7%); colorless oil; $[\alpha]_D^{25}$ +127.7 (*c* 0.68, $CHCl_3$); ^1H NMR (500 MHz): δ 2.02-2.09 (m,2H), 2.37 (broad s, 1H), 3.45 and 3.48 (two d, *J* = 6.5 and 6.0 Hz, 1H), 3.56 and 3.58 (two d, *J* = 3.5 Hz both, 1H), 3.85 (broad s, 1H), 3.91-3.93 (m, 1H), 4.27 (d, *J* = 11.5 Hz, 1H), 4.47 (d, *J* = 11.5 Hz, 1H), 4.63 and 4.65 (two d, *J* = 3.5 Hz both, 1H), 7.28-7.41 (m, 10H); ^{13}C NMR (125 MHz): δ 41.4, 66.6, 70.5, 71.6, 81.5, 126.6, 127.9, 128.0, 128.1, 128.5, 128.7, 137.5, 141.2.

(2*S*,4*S*)-4-Phenylbutane-1,2,4-triol (7a').[6] To a solution of **7a** (25 mg, 0.092 mmol) in 80% (v/v) aqueous CH_2Cl_2 (2 mL) was added DDQ (31 mg, 0.138 mmol) at room temperature. After stirring for 3 h (*cf*. TLC, 5% $CH_3OH/CHCl_3$), the reaction mixture was quenched with saturated aqueous $NaHCO_3$ and extracted with CH_2Cl_2 (1 × 5 mL) followed by Et_2O (2 × 2 mL). The combined organic extract was washed with water (2 × 5 mL) and brine (1 ×

3 mL), and dried. Removal of the solvent in vacuo followed by column chromatography (silica gel, 0–5% $CH_3OH/CHCl_3$) of the residue afforded pure **7a'**. Yield: 13 mg, (78%); colorless oil; $[\alpha]_D^{22}$: -20.9 (*c* 0.60, $CHCl_3$) [lit.[5] $[\alpha]_D$: -21.3 (*c* 0.40, CH_2Cl_2)]; IR (film): ν_{max} 3098 cm^{-1}; ^{1}H NMR (200 MHz): δ 1.73 (broad s, 3H), 1.84-1.93 (m, 2H), 3.60-3.64 (m, 1H), 3.66-3.70 (m, 1H), 3.91-4.02 (m, 1H), 5.05 (dd, *J* = 4.2 and 7.6 Hz, 1H), 7.25-7.36 (m, 5H); ^{13}C NMR (50 MHz): δ 41.0, 66.7, 69.4, 71.6, 125.4, 127.5, 128.5, 144.1.

(2*S*,4*S*)-4-(Benzyloxy)-2-hydroxy-4-phenylbutyl benzoate (8). Et_3N (0.74 mL, 5.29 mmol) and BzCN (636 mg, 4.85 mmol) were added to a cooled (0 °C) and stirred solution of **7a** (1.20 g, 4.41 mmol) in CH_2Cl_2 (20 mL). The mixture was further stirred at 0 °C till completion of the reaction (*cf.* TLC, 10% EtOAc/hexane, 2 h). Then the reaction was quenched with H_2O (10 mL), and extracted with $CHCl_3$ (3 × 10mL). The combined organic extract was washed with water (3 × 10 mL) and brine (1 × 5 mL), and dried. Removal of solvent in vacuo followed by column chromatography (silica gel, 0–10% EtOAc/hexane) of the residue afforded pure **8**. Yield: 1.46 g (88%); colorless oil; $[\alpha]_D^{22}$: -41.7 (*c* 1.16, $CHCl_3$); IR (film): ν_{max} 3471, 3063, 3031, 1717, 1275 cm^{-1}; ^{1}H NMR (500 MHz): δ 1.90-1.95 (m,1H), 2.03-2.08 (m, 1H), 2.86 (broad s, 1H), 4.26-4.33 (m, 3H), 4.37 and 4.39 (two d, *J* = 3.0 and 2.5 Hz, 1H), 4.51 (d, *J* = 11.5 Hz, 1H), 4.75 and 4.78 (two d, *J* = 3.0 and 2.5 Hz, 1H), 7.28-7.37 (m, 6H), 7.39-7.45 (m, 6H), 7.55-7.58 (m, 1H), 8.04 (d, *J* = 7.5 Hz, 2H); ^{13}C NMR (125 MHz): δ 41.6, 67.1, 68.8, 70.8, 78.1, 126.5, 126.7, 127.8, 127.9, 128.0, 128.3, 128.4, 128.5, 128.7, 128.8, 129.7, 133.1, 138.0, 141.6, 166.6. Anal. Calcd. for $C_{24}H_{24}O_4$: C, 76.57; H, 6.43%. Found: C, 76.46; H, 6.66%.

(2*R*,4*S*)-2-Azido-4-(benzyloxy)-4-phenylbutyl benzoate (9). Et_3N (0.75 mL, 5.34 mmol) and $MeSO_2Cl$ (0.47 mL, 6.05 mmol) were added to a solution of **8** (1.34 g, 3.56 mmol) in CH_2Cl_2 (5 mL) at 0 °C. After stirring the reaction mixture for another 0.5 h at 0 °C, the mixture was brought to room temperature, and stirred for additional 2 h. After completion of the reaction (*cf.* TLC, 10% EtOAc/hexane,), the mixture was quenched with H_2O (10 mL) and extracted with $CHCl_3$ (3 × 10 mL). The combined organic extract was washed with H_2O (2 × 10 mL), brine (1 × 5 mL), and dried. Solvent removal afforded the corresponding mesylate which was used as such in the next step.

The crude mesylate was dissolved in DMF (5 mL) and NaN_3 (579 mg, 8.90 mmol) was added. The mixture was heated at 90 °C till completion of the reaction (*cf.* TLC, 10% EtOAc/hexane, 3 h). After concentrating the reaction mixture in vacuo, the residue was diluted with H_2O (30 mL) and extracted with EtOAc (3 × 20 mL). The combined organic extracts were washed successively with H_2O (2 × 10 mL) and brine (1 × 5 mL), and dried. Removal of solvent in vacuo followed by column chromatography (silica gel, 0–10% EtOAc/hexane) of the residue afforded pure **9**. Yield: (1.14 g, 80% in two steps); colorless oil; $[\alpha]_D^{22}$ -23.9 (*c* 0.92, $CHCl_3$); IR (film): ν_{max} 3063, 3031, 2105, 1723, 1271 cm^{-1}; ^{1}H NMR (500 MHz): δ 1.91-1.96 (m,1H), 2.19-2.25 (m, 1H), 3.65-3.68 (m, 1H), 4.26-4.34 (m, 2H), 4.42-4.46 (m, 2H), 4.55 (t, *J* = 7.0 Hz, 1H), 7.29-7.47 (m, 12H), 7.56-7.60 (m, 1H), 8.04 (d, *J* = 8.0 Hz, 2H); ^{13}C NMR (125 MHz): δ 38.9, 58.1, 66.9, 70.5, 78.1, 126.9, 127.7, 127.9, 128.3, 128.4, 128.5, 128.8, 129.8, 133.3, 137.9, 140.7, 166.1. Anal. Calcd. for $C_{24}H_{23}N_3O_3$: C, 71.80; H, 5.77; N, 10.47%. Found: C, 71.79; H, 5.52; N, 10.49%.

***N*-((2*R*,4*S*)-4-(Benzyloxy)-1-hydroxy-4-phenylbutan-2-yl)dodecanamide (9a).** To a stirred suspension of $LiAlH_4$ (47 mg, 1.25 mmol) in anhydrous THF (5 mL) was added a solution of

9 (100 mg, 0.25 mmol) in THF (2 mL), and the mixture stirred vigorously at room temperature. After 3 h (*cf.* TLC, 10% $CH_3OH/CHCl_3$) the reaction was quenched with saturated aqueous Na_2SO_4 and passed through a pad of silica gel (eluted by EtOAc). Removal of solvent in vacuo yielded the amino alcohol, which was used in the next step without further purification.

A solution of the crude amino alcohol in CH_2Cl_2 (5 mL) was added to the mixture of DCC (58 mg, 0.28 mmol), DMAP (catalytic) and lauric acid (52 mg, 0.26 mmol) in CH_2Cl_2 (5 mL) and the reaction mixture was stirred for 24 h (*cf.* TLC, 3% $MeOH/CHCl_3$). Then, CH_2Cl_2 was removed in vacuo, the residue diluted with EtOAc (10 mL), filtered and concentrated in vacuo. The residue was purified by column chromatography (silica gel, 0–3% $MeOH/CHCl_3$) to get pure **9a**. Yield: (68 mg, 60% in two steps); colorless oil; $[\alpha]_D^{25}$-48.5 (*c* 1.20, $CHCl_3$); IR (film): ν_{max} 3025, 1640 cm^{-1};^{1}H NMR (500 MHz): δ 0.89 (t, *J* = 7.0 Hz, 3H), 1.24-1.32 (m, 16H), 1.50-1.53 (m, 2H), 1.96-2.04 (m, 5H), 3.57 (d, *J* = 3.0 Hz, 2H), 4.01-4.02 (m, 1H), 4.19-4.22 (m, 1H), 4.42-4.48 (m, 2H), 6.41 (d, *J* = 4.5 Hz, 1H), 7.26-7.40 (m, 10H); ^{13}C NMR (125 MHz): δ 14.1, 22.7, 25.7, 29.3, 29.5, 29.6, 31.9, 36.7, 39.6, 51.8, 66.5, 70.6, 79.8, 126.5, 127.9, 128.0, 128.1, 128.5, 128.8, 137.6, 141.4, 174.4. Anal. Calcd. for $C_{29}H_{43}NO_3$: C, 76.78; H, 9.55; N, 3.09%. Found: C, 76.89; H, 9.71; N, 3.18%.

***N*-((*R*)-1-Hydroxy-4-phenylbutan-2-yl)dodecanamide (9b)**. A mixture of **9a** (25 mg, 0.06 mmol) and 10% Pd–C (5 mg) in EtOH (10 mL) was stirred under a positive pressure of H_2 gas for 18 h. The reaction mixture was diluted with ether, passed through a pad of silica gel and the eluent concentrated in vacuo to give pure **9b**. Yield: (11 mg, 54%); white solid; $[\alpha]_D^{25}$ -38.5 (*c* 0.80, $CHCl_3$); IR (film): ν_{max} 2980, 1642 cm^{-1}; ^{1}H NMR (500 MHz): δ 0.89 (t, *J* = 7.0 Hz, 3H), 1.26-1.33 (m, 16H), 1.61-1.63 (m, 3H), 1.80-1.83 (m, 1H), 1.88-1.91 (m, 1H), 2.14-2.18 (m, 2H), 2.67-2.70 (m, 2H), 3.57-3.61 (m, 1H), 3.66-3.70 (m, 1H), 3.98-4.00 (m, 1H), 5.76 (d, *J* = 8.0 Hz, 1H), 7.17-7.21 (m, 3H), 7.27-7.30 (m, 2H); ^{13}C NMR (125 MHz): δ 14.1, 22.7, 25.8, 29.3, 29.4, 29.5, 29.6, 31.9, 32.5, 32.8, 36.9, 51.6, 65.6, 126.1, 128.3, 128.5, 141.4, 174.2.

(2*R*,4*S*)-2-Azido-4-hydroxy-4-phenylbutyl benzoate (10). To a solution of the **9** (1.0 g, 2.49 mmol) in 80% (v/v) aqueous CH_2Cl_2 (15 mL) was added DDQ (734 mg, 3.24 mmol) at room temperature. After stirring for 3 h (*cf.* TLC, 15% EtOAc/hexane), the reaction was quenched with saturated aqueous $NaHCO_3$ and extracted with CH_2Cl_2 (1 × 10 mL) followed by diethyl ether (2 × 10 mL). The combined organic extract was washed with water (2 × 10 mL) and brine (1 × 5 mL), and dried. Removal of solvent in vacuo followed by column chromatography (silica gel, 0–15% EtOAc/hexane) of the residue afforded pure **10**. Yield: 651 mg (84%); colorless oil; $[\alpha]_D^{25}$ -12.0 (*c* 0.70, $CHCl_3$); IR (film): ν_{max} 3423, 2106, 1722, 1272 cm^{-1};^{1}H NMR (500 MHz): δ 1.92-1.97 (m, 1H), 2.08-2.14 (m, 1H), 2.32 (broad s, 1H), 3.72-3.77 (m, 1H), 4.38 and 4.40 (two d, *J* = 7.5 Hz both, 1H), 4.52 and 4.54 two d, *J* = 3.5 and 3.0 Hz, 1H), 4.92 and 4.93 (two d, *J* = 6.0 Hz both, 1H), 7.31-7.33 (m, 1H), 7.38-7.39 (m, 4H), 7.44-7.47 (m, 2H), 7.56-7.59 (m, 1H), 8.05 (d, *J* = 7.0 Hz, 2H); ^{13}C NMR (125 MHz): δ 39.6, 58.7, 67.0, 71.9, 125.9, 128.2, 128.5, 128.8, 129.4, 129.8, 132.1, 133.3, 143.3, 166.2. Anal. Calcd. for $C_{17}H_{17}N_3O_3$: C, 65.58; H, 5.50; N, 13.50%. Found: C, 65.59; H, 5.63; N, 13.52%.

(1*R*,3*S*)-*N*-(3-Hydroxy-1-hydroxymethyl-3-phenylprop-1-yl)dodecan-amide (2).[7] As per Scheme 3, oxidative debenzylation of **9a** (25 mg, 0.06 mmol) using DDQ (method as

described before), and subsequent purification by column chromatography (silica gel, 0–10% MeOH/$CHCl_3$) yielded pure **2** (yield 3.7 mg, 17%).

Alternatively, as per Scheme 4, to a stirred suspension of LAH (307 mg, 8.08 mmol) in anhydrous THF (10 mL) was added a solution of **10** (630 mg, 2.02 mmol) in THF (2 mL), and the mixture stirred vigorously at room temperature. After 3 h the reaction was quenched with saturated aqueous Na_2SO_4 and passed through a pad of silica gel (eluted with EtOAc). Removal of solvent in vacuo gave the aminodiol, which was used for next reaction without further purification.

A solution of the crude aminodiol in CH_2Cl_2 (10 mL) was added to the mixture of DCC (501 mg, 2.42 mmol), DMAP (catalytic) and lauric acid (404 mg, 2.02 mmol) in CH_2Cl_2 (10 mL) and the mixture stirred for 18 h (*cf.* TLC, 10% MeOH/$CHCl_3$). Then, CH_2Cl_2 was removed under vacuo, the residue diluted with EtOAc (20 mL), filtered and concentrated in vacuo. The residue was purified by column chromatography (silica gel, 0–10% MeOH/$CHCl_3$) to afford **2**. Yield: 462 mg (63% in two steps); white solid; mp 88-89 ^{o}C; lit.[6] mp 86-88 ^{o}C; $[\alpha]_D^{25}$ -32.4 (*c* 1.1, $CHCl_3$); lit.[6] $[\alpha]_D^{25}$ -30.7 (*c* 0.533, $CHCl_3$); IR (KBr): ν_{max} 2922, 1641 cm^{-1}; ^1H NMR (200 MHz): δ 0.88 (t, J = 6.6 Hz, 3H), 1.24-1.39 (m, 16H), 1.54-1.63 (m, 2H), 1.93-2.04 (m, 2H), 2.17 (t, J = 8.0 Hz, 2H), 2.32 (t, J = 7.4 Hz, 2H), 3.66–3.68 (m, 2H), 4.06-4.09 (m, 1H), 4.78-4.84 (m, 1H), 6.46 (d, J = 5.8 Hz, 1H), 7.28–7.35 (m, 5H); ^{13}C NMR (50 MHz): δ 14.1, 22.7, 24.8, 25.7, 29.1, 29.4, 29.5, 29.6, 31.9, 36.8, 40.7, 50.5, 65.9, 72.0, 125.6, 127.8, 128.6, 144.2, 174.6.

References

1. Dey, P.; Koli, M.; Goswami, D.; Sharma, A.; Chattopadhyay, S. *Eur. J. Org. Chem.* **2018**, 1333-1341.
2. Bracher, F.; Litz, T. *Bioorg. Med. Chem.***1996**, 4, 877-880.
3. Rauniyar, V.; Zhai, H.; Hall, D. G. *J. Am. Chem. Soc*. **2008**, *130*, 8481–8490.
4. Brown, H. C.; Kulkarni, S. V.; Racherla, U. S. *J. Org. Chem.* **1994**,*59*, 365-369.
5. Cossrow, J.; Rychnovsky, S. D. *Org. Lett.* **2002**, *4*, 147–150.
6. Bachki, A.; Foubelo, F.; Yus, M. *Tetrahedron: Asymmetry* **1996**, *7*, 2997-3008.
7. Chacko, S.; Kalita, M.; Ramapanicker, R. *Tetrahedron: Asymmetry* **2015**, *26*, 623–631.
