# Supplementary material for: A chemoenzymatic synthesis of ceramide trafficking inhibitor HPA-12
Source: Beilstein J Org Chem. 2019 Feb 18;15:490–6. doi: 10.3762/bjoc.15.42 (PMC6404422; doi:10.3762/bjoc.15.42)
Supplement: File 2 — NMR spectra. [file Beilstein_J_Org_Chem-15-490-s002.pdf]

BEILSTEIN JOURNAL OF ORGANIC CHEMISTRY

Supporting Information

for

A chemoenzymatic synthesis of ceramide trafficking inhibitor HPA-12

Seema V. Kanojia, Sucheta Chatterjee, Subrata Chattopadhyay and Dibakar Goswami

Beilstein J. Org. Chem. **2019,** *15,* 490–496. doi:10.3762/bjoc.15.42

NMR spectra

License and Terms: This is a supporting information file under the terms of the Creative Commons Attribution License (http://creativecommons.org/licenses/by/4.0). Please note that the reuse, redistribution and reproduction in particular requires that the authors and source are credited.
The license is subject to the *Beilstein Journal of Organic Chemistry* terms and conditions: (https://www.beilstein-journals.org/bjoc)

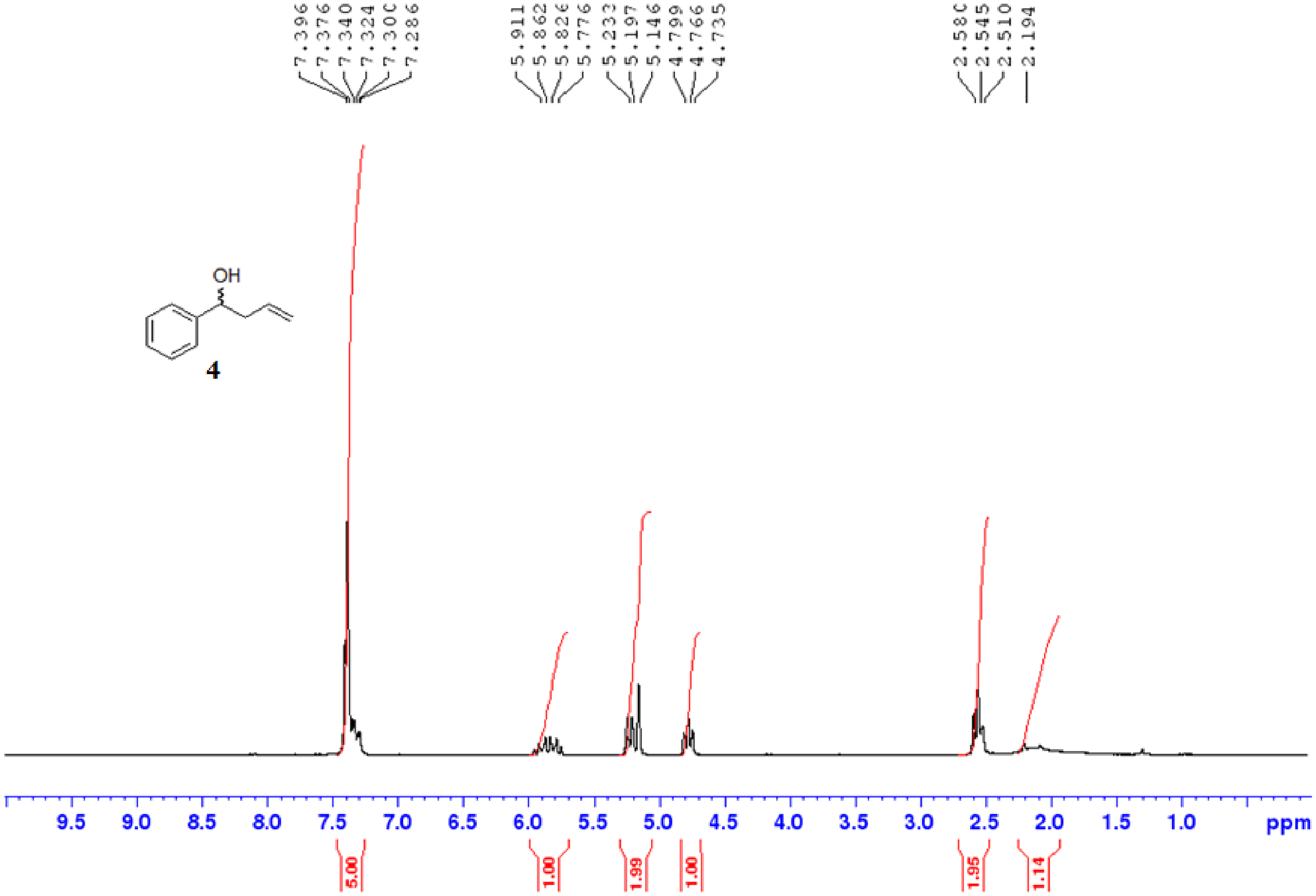

7.396
7.376
7.340
7.324
7.300
7.286
5.911
5.862
5.826
5.776
5.233
5.197
5.146
4.799
4.766
4.735
2.580
2.545
2.510
2.194
OH
4
9.5
9.0
8.5
8.0
7.5
7.0
6.5
6.0
5.5
5.0
4.5
4.0
3.5
3.0
2.5
2.0
1.5
1.0
ppm
5.00
1.00
1.99
1.00
1.95
1.14

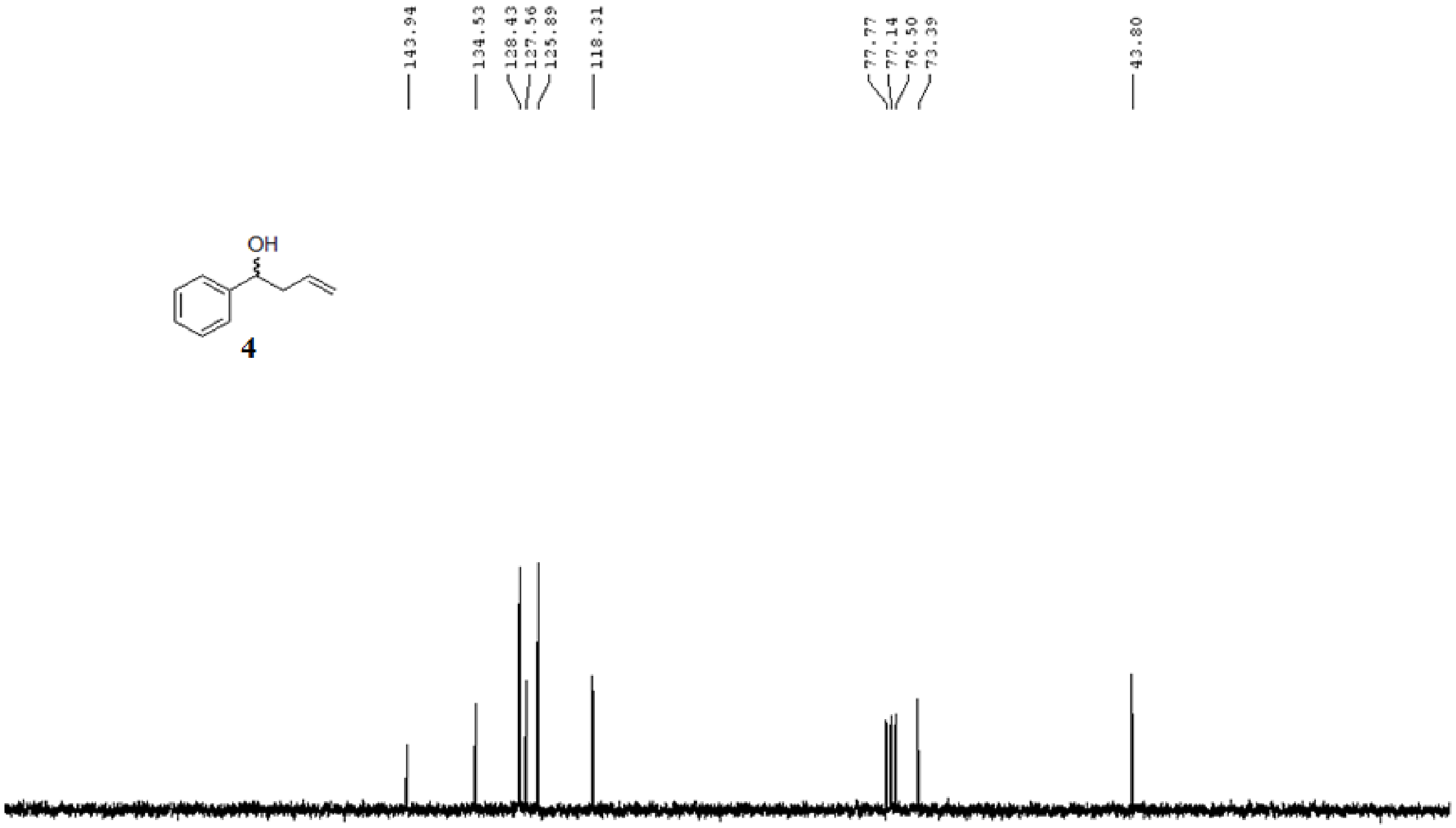
143.94
134.53
128.43
127.56
125.89
118.31
77.77
77.14
76.50
73.39
43.80
OH
4

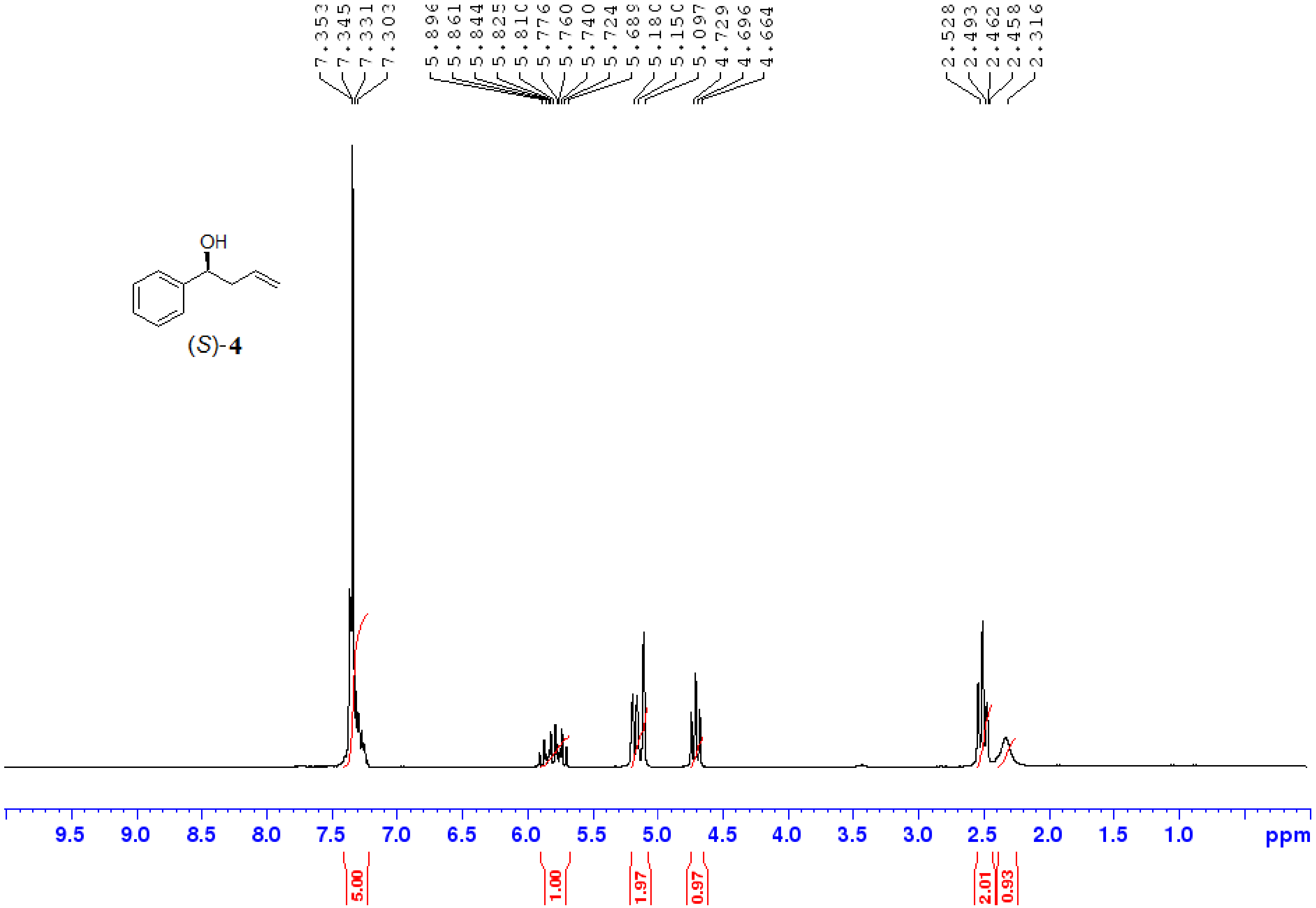
OH
7.353
7.345
7.331
7.303
5.896
5.861
5.844
5.825
5.810
5.776
5.760
5.740
5.724
5.689
5.180
5.150
5.097
4.729
4.696
4.664
2.528
2.493
2.462
2.458
2.316
9.5
9.0
8.5
8.0
7.5
7.0
6.5
6.0
5.5
5.0
4.5
4.0
3.5
3.0
2.5
2.0
1.5
1.0
ppm
5.00
1.00
1.97
0.97
2.01
0.93

(*S*)-**4**

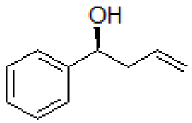

(*S*)-**4**

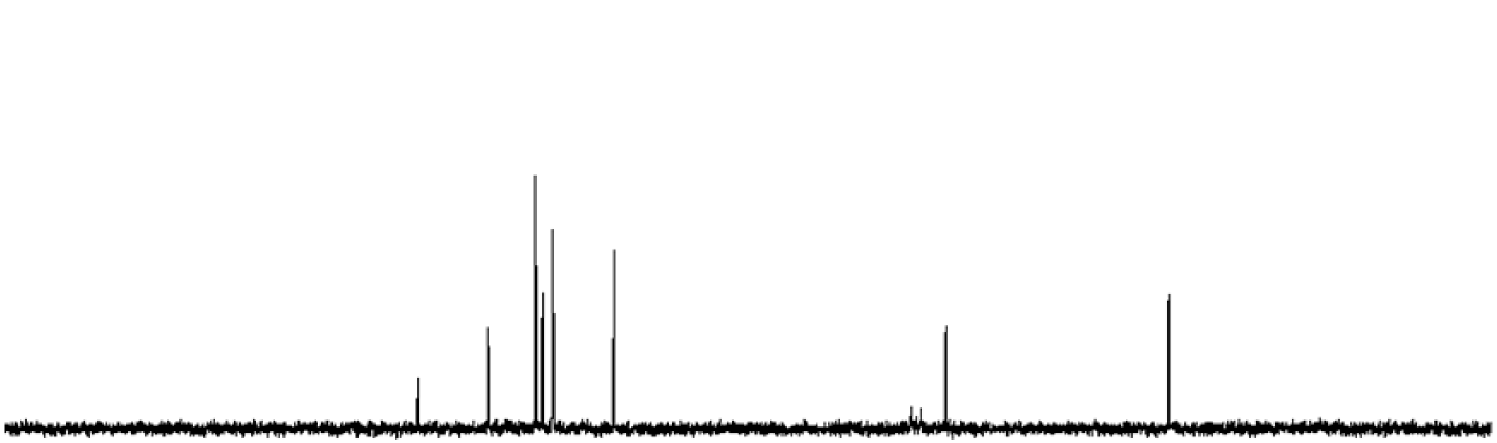

143.72
134.30
127.96
127.06
125.64
117.47
77.65
77.00
76.38
73.11
43.29

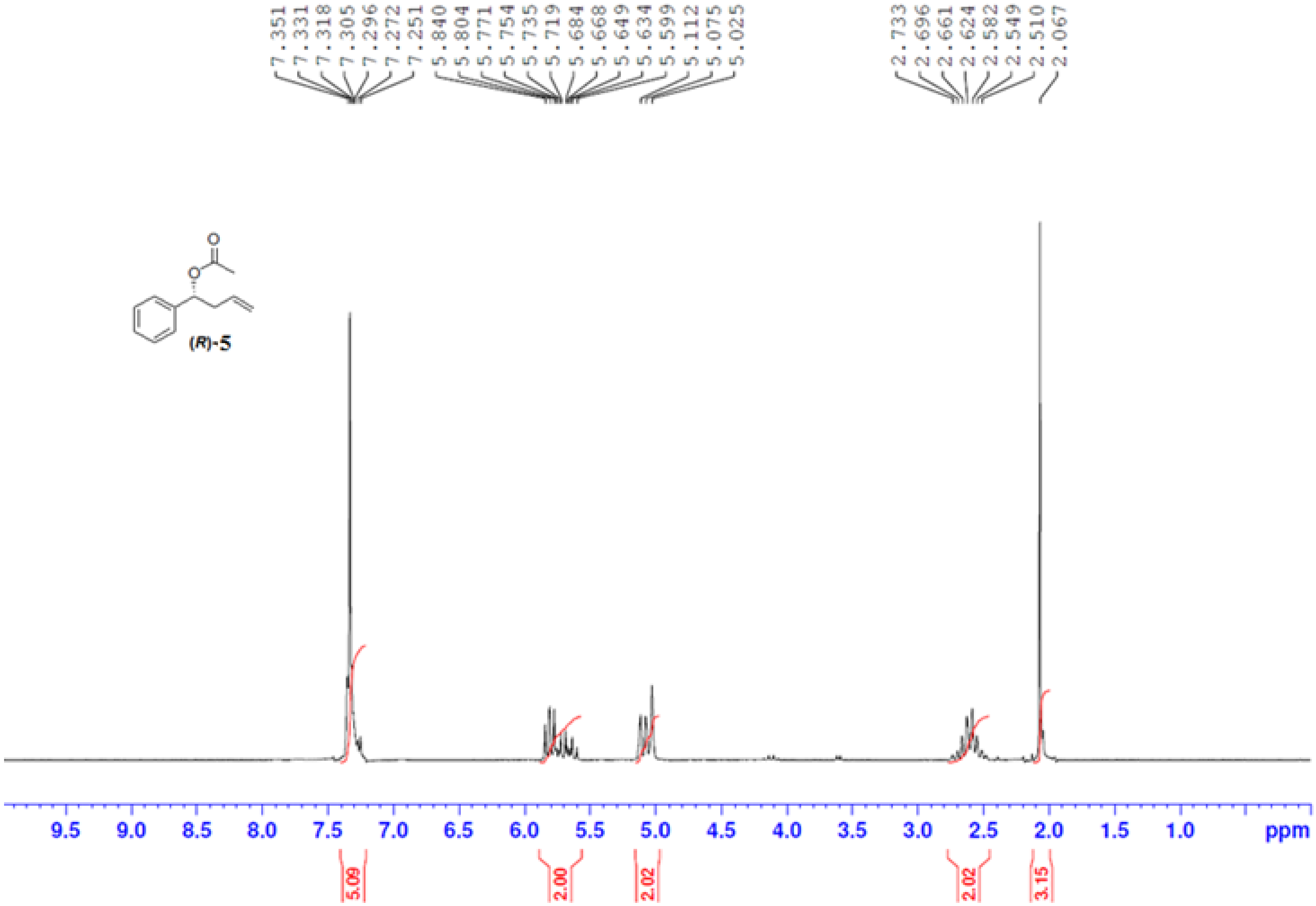

7.351
7.331
7.318
7.305
7.296
7.272
7.251
5.840
5.804
5.771
5.754
5.735
5.719
5.684
5.668
5.649
5.634
5.599
5.112
5.075
5.025
2.733
2.696
2.661
2.624
2.582
2.549
2.510
2.067
(R)-5
9.5
9.0
8.5
8.0
7.5
7.0
6.5
6.0
5.5
5.0
4.5
4.0
3.5
3.0
2.5
2.0
1.5
1.0
ppm
5.09
2.00
2.02
2.02
3.15

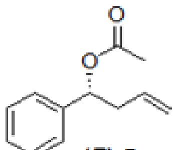
O
O

(*R*)-5

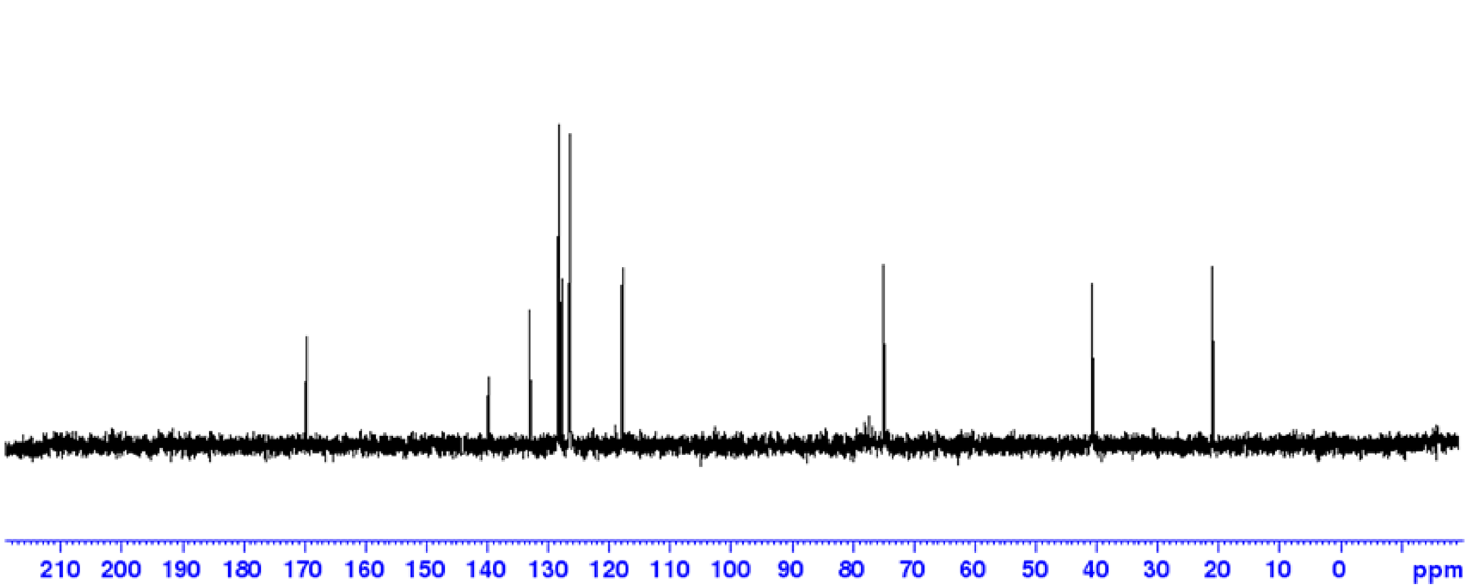
169.46
139.72
132.96
127.97
127.48
126.13
117.53
77.94
76.88
76.40
74.59
40.32
20.56
210 200 190 180 170 160 150 140 130 120 110 100 90 80 70 60 50 40 30 20 10 0 ppm

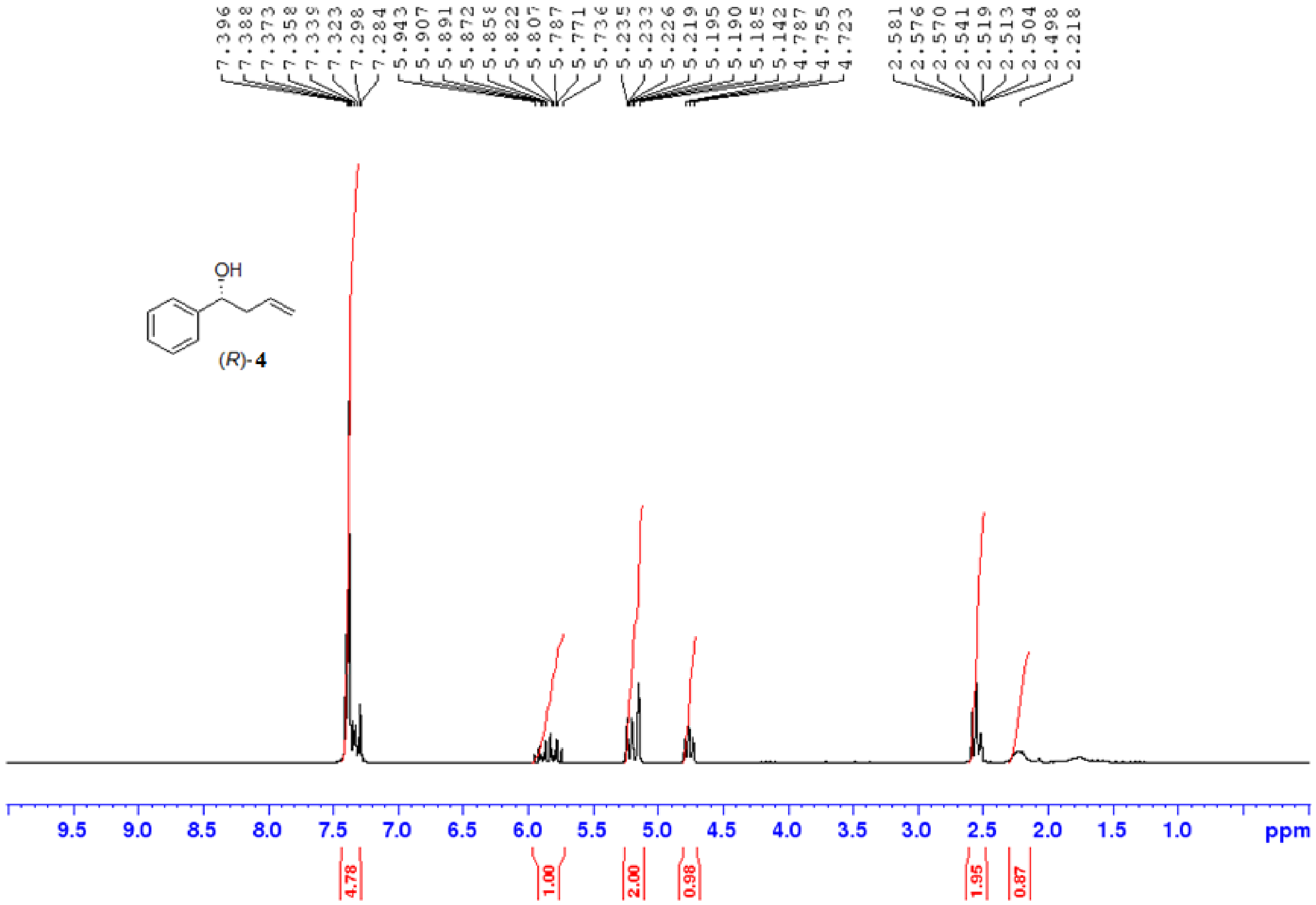
7.396
7.388
7.373
7.358
7.339
7.323
7.298
7.284
5.943
5.907
5.891
5.872
5.858
5.822
5.807
5.787
5.771
5.736
5.235
5.233
5.226
5.219
5.195
5.190
5.185
5.142
4.787
4.755
4.723
2.581
2.576
2.570
2.541
2.519
2.513
2.504
2.498
2.218
OH
(R)-4
9.5
9.0
8.5
8.0
7.5
7.0
6.5
6.0
5.5
5.0
4.5
4.0
3.5
3.0
2.5
2.0
1.5
1.0
ppm
4.78
1.00
2.00
0.98
1.95
0.87

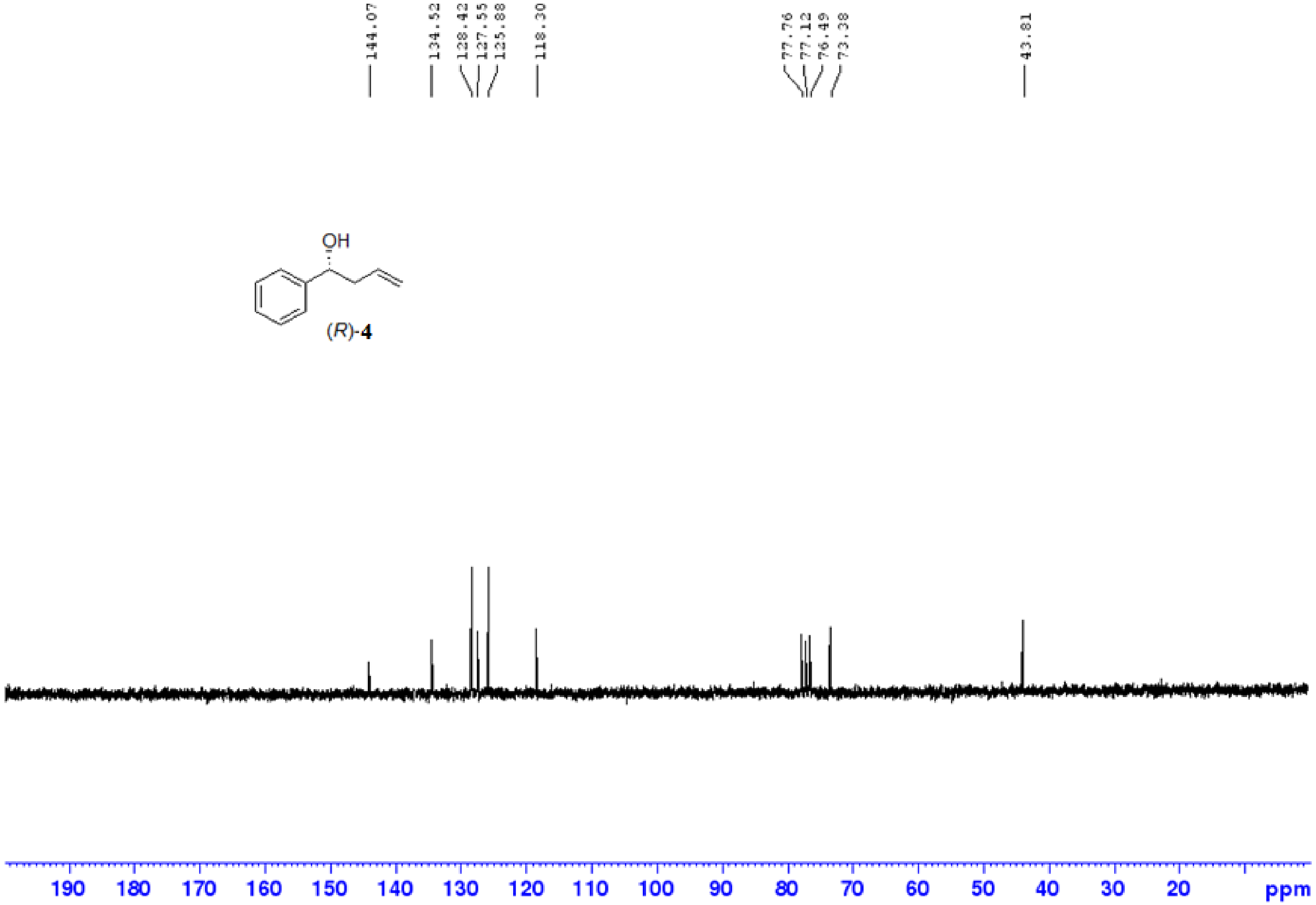
144.07
134.52
128.42
127.55
125.88
118.30
77.76
77.12
76.49
73.38
43.81
OH
(R)-4
190 180 170 160 150 140 130 120 110 100 90 80 70 60 50 40 30 20
ppm

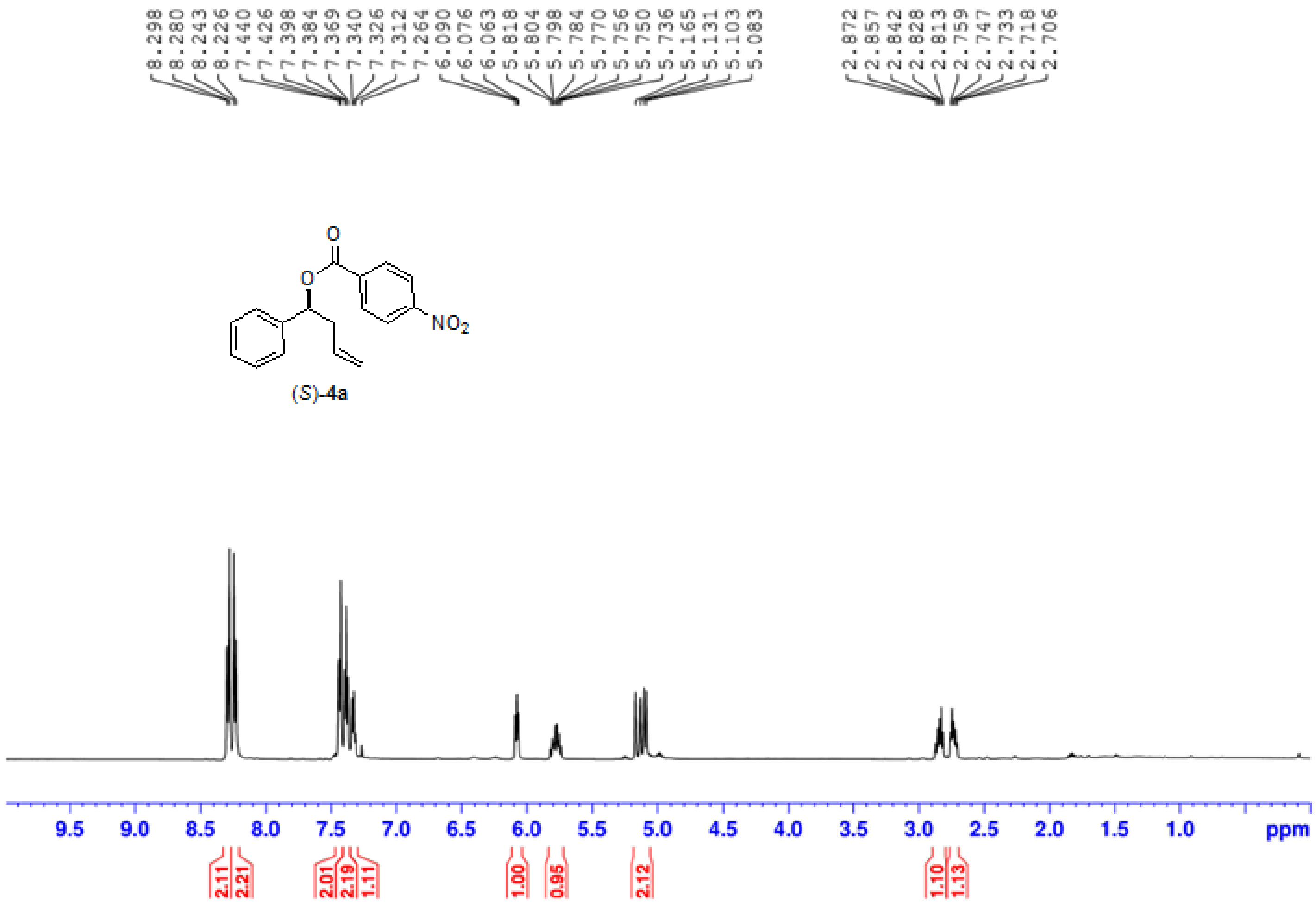
8.298
8.280
8.243
8.226
7.440
7.426
7.398
7.384
7.369
7.340
7.326
7.312
7.264
6.090
6.076
6.063
5.818
5.804
5.798
5.784
5.770
5.756
5.750
5.736
5.165
5.131
5.103
5.083
2.872
2.857
2.842
2.828
2.813
2.759
2.747
2.733
2.718
2.706
O
O
NO_2
(S)-4a
9.5
9.0
8.5
8.0
7.5
7.0
6.5
6.0
5.5
5.0
4.5
4.0
3.5
3.0
2.5
2.0
1.5
1.0
ppm
2.11
2.21
2.01
2.19
1.11
1.00
0.95
2.12
1.10
1.13

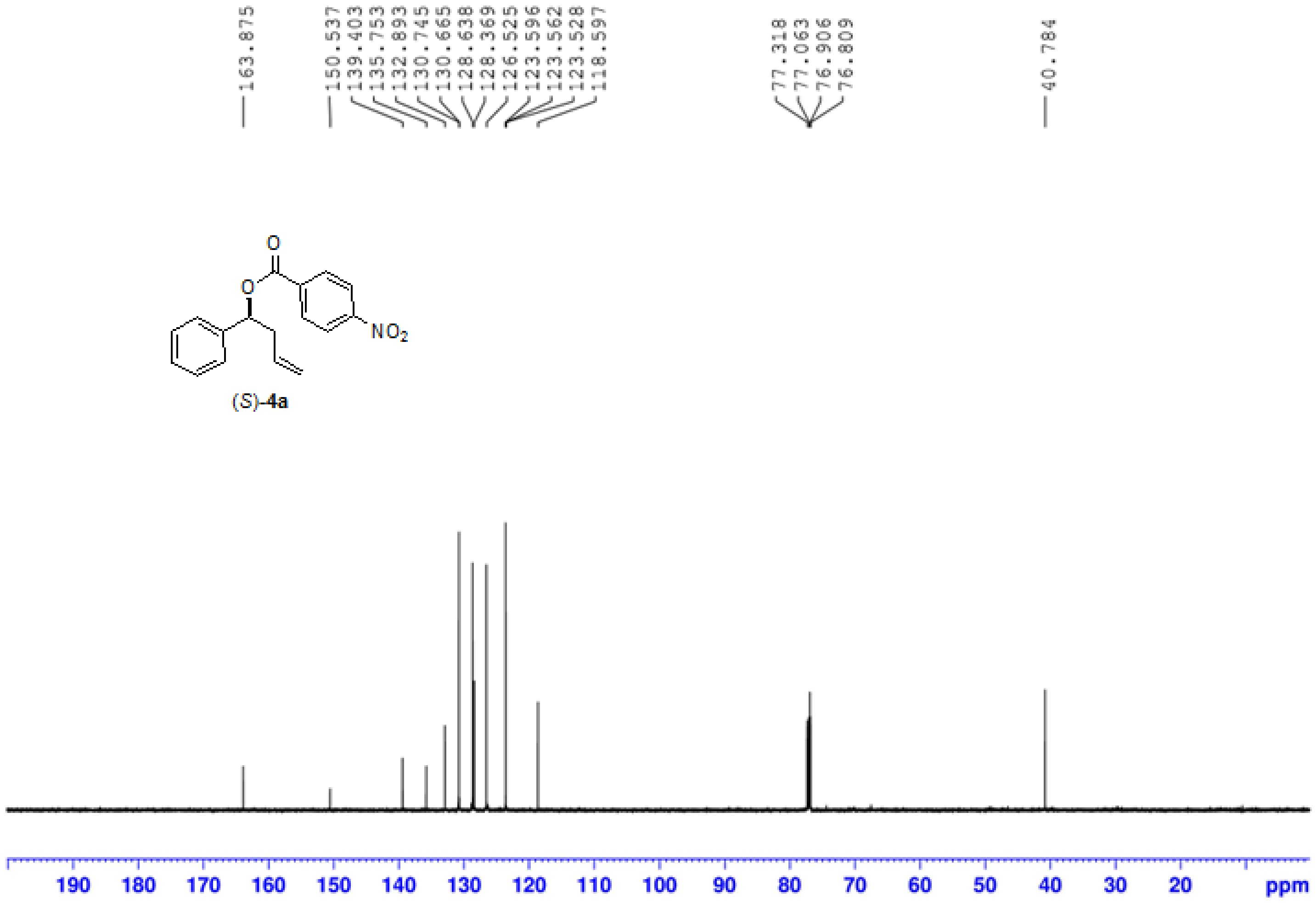
163.875
150.537
139.403
135.753
132.893
130.745
130.665
128.638
128.369
126.525
123.596
123.562
123.528
118.597
77.318
77.063
76.906
76.809
40.784
O
O
NO2
(S)-4a
190
180
170
160
150
140
130
120
110
100
90
80
70
60
50
40
30
20
ppm

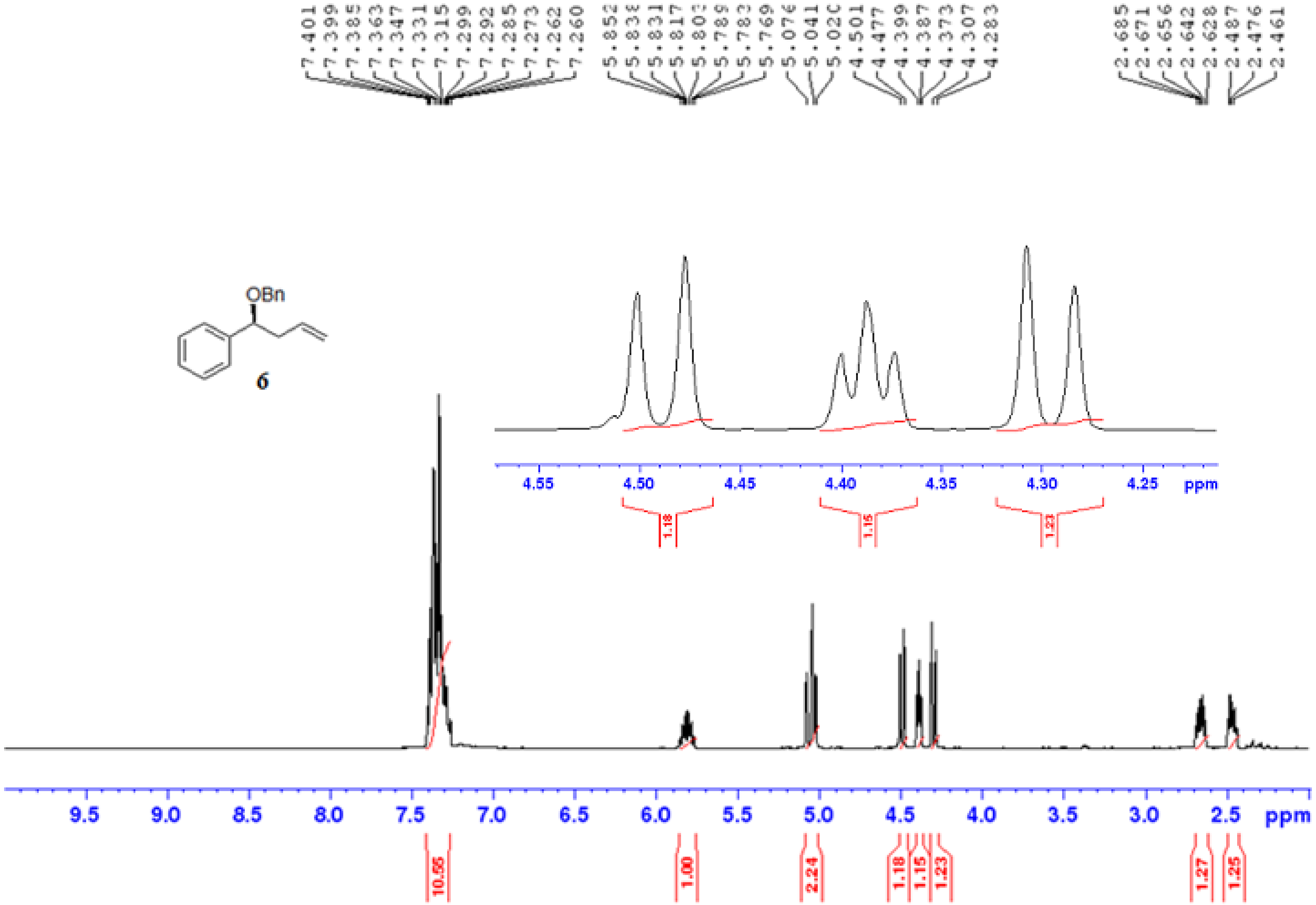
OBn
ppm
9.5
9.0
8.5
8.0
7.5
7.0
6.5
6.0
5.5
5.0
4.5
4.0
3.5
3.0
2.5
4.55
4.50
4.45
4.40
4.35
4.30
4.25
10.55
1.00
2.24
1.18
1.15
1.23
1.27
1.25

6

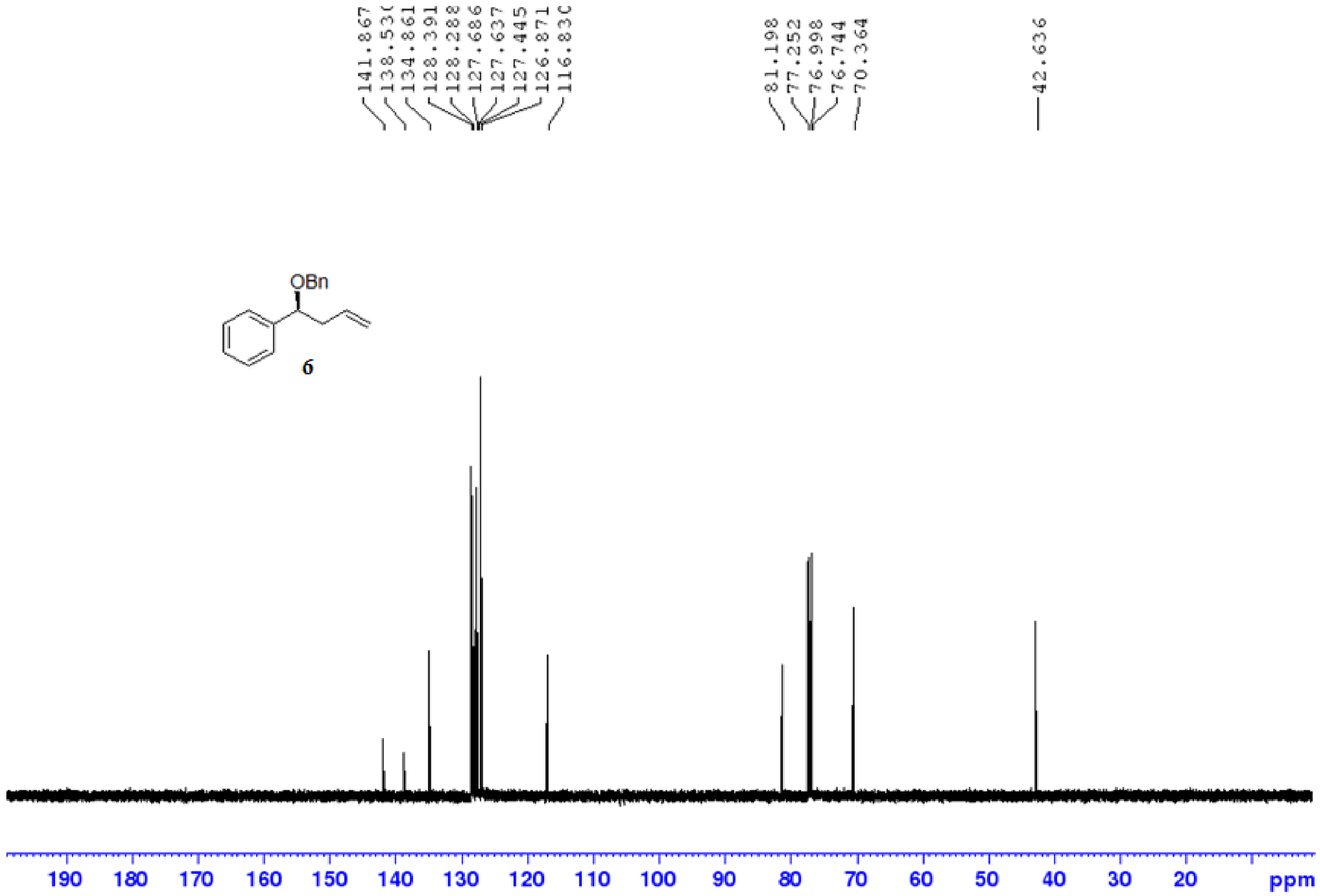
141.867
138.53(
134.861
128.391
128.288
127.686
127.637
127.445
126.871
116.83(
81.198
77.252
76.998
76.744
70.364
42.636
OBn
6
190 180 170 160 150 140 130 120 110 100 90 80 70 60 50 40 30 20 ppm

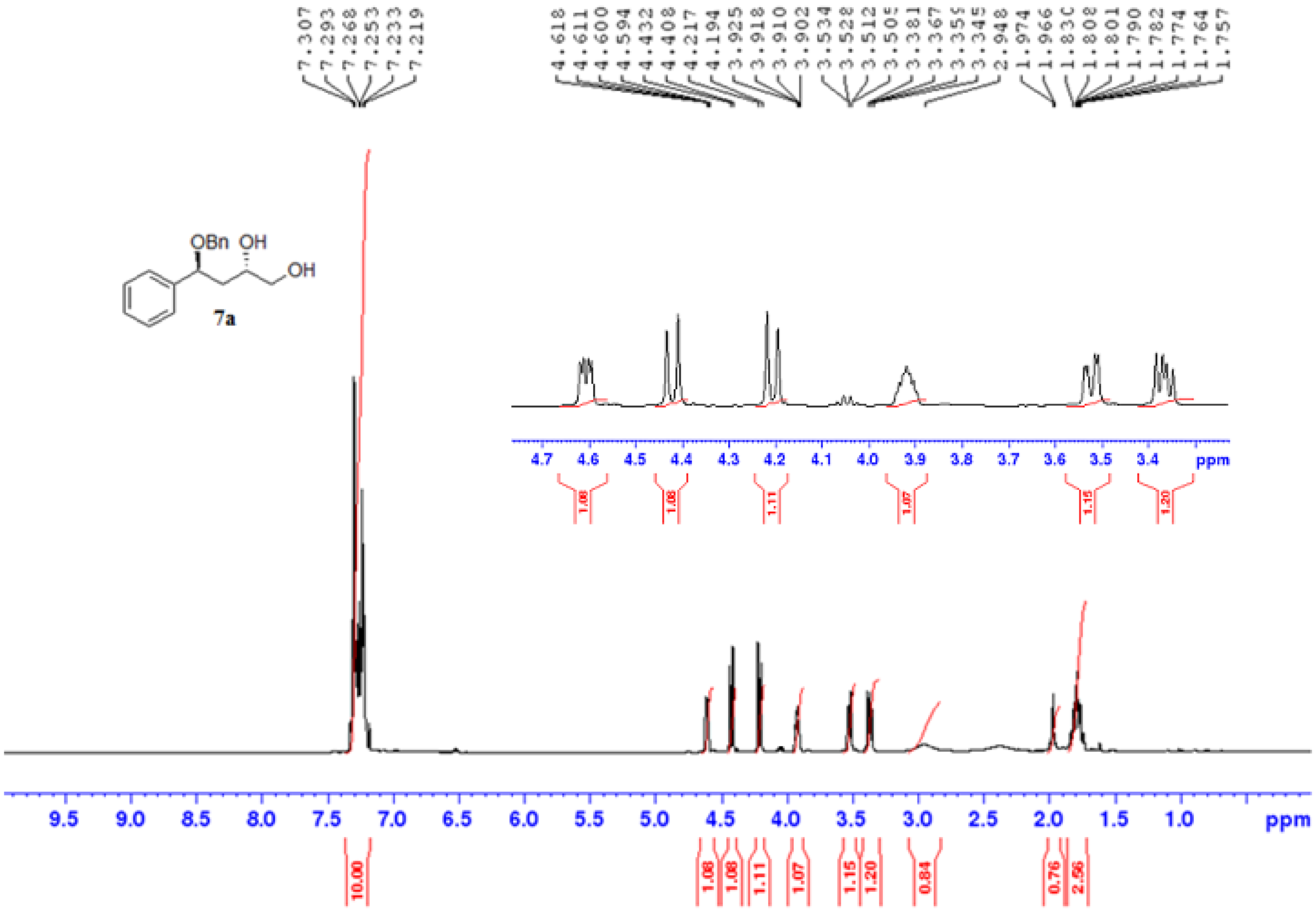
OBn OH
OH
7a
ppm

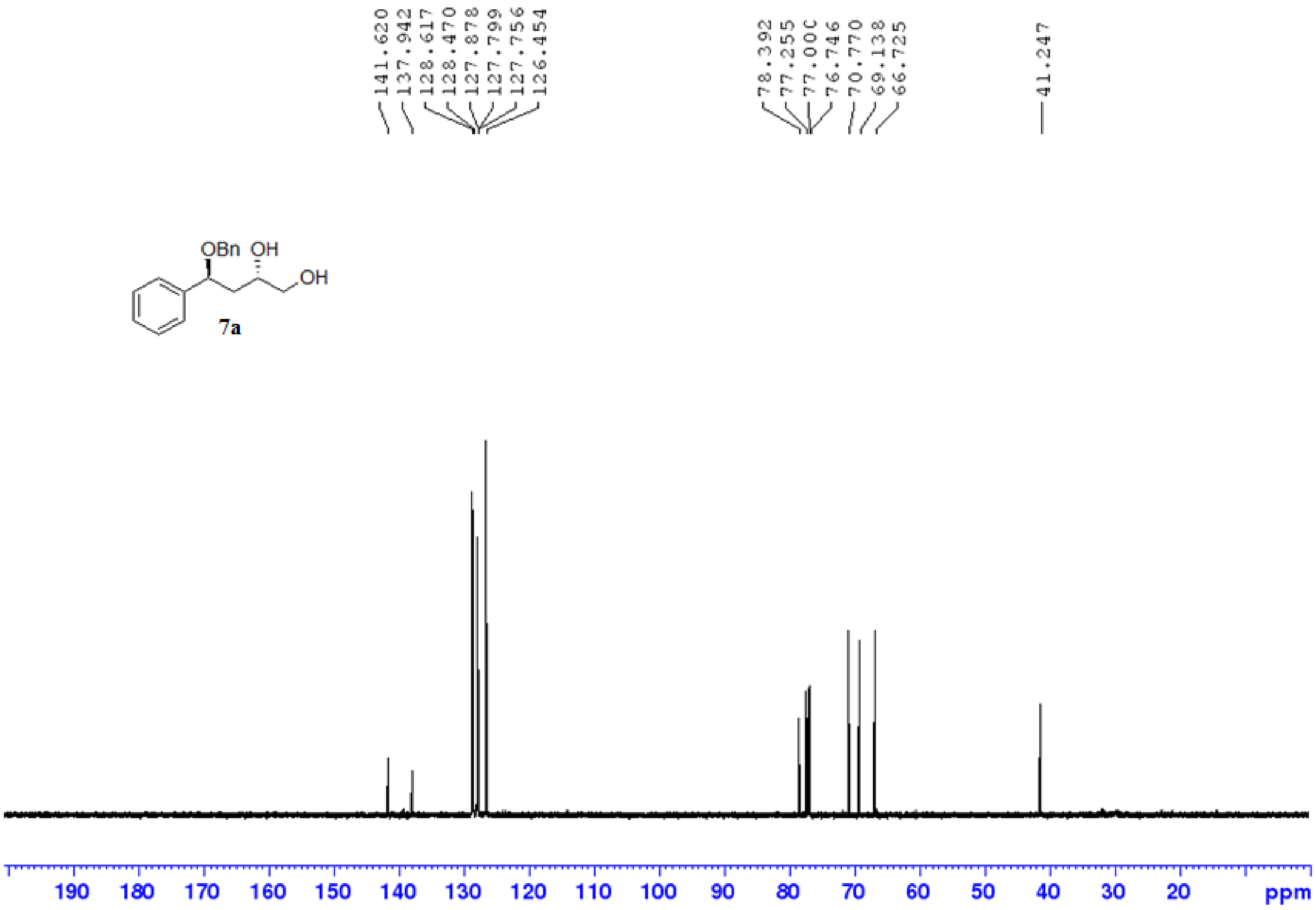

141.620
137.942
128.617
128.470
127.878
127.799
127.756
126.454
78.392
77.255
77.00C
76.746
70.770
69.138
66.725
41.247
OBn
OH
OH
7a
190
180
170
160
150
140
130
120
110
100
90
80
70
60
50
40
30
20
ppm

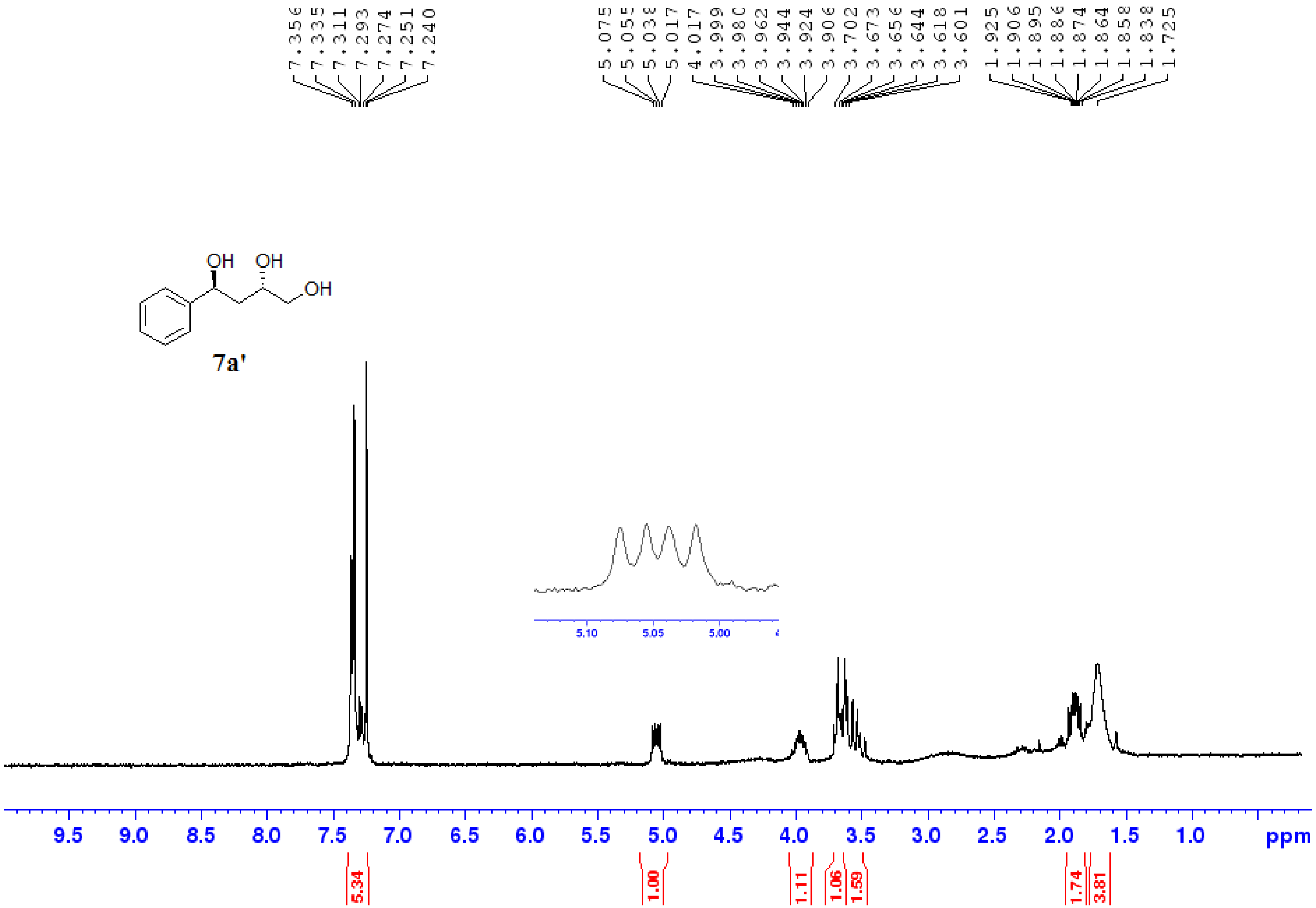
7.356
7.335
7.311
7.293
7.274
7.251
7.240
5.075
5.055
5.038
5.017
4.017
3.999
3.980
3.962
3.944
3.924
3.906
3.702
3.673
3.656
3.644
3.618
3.601
1.925
1.906
1.895
1.886
1.874
1.864
1.858
1.838
1.725
OH
OH
OH
7a'
5.10
5.05
5.00
9.5
9.0
8.5
8.0
7.5
7.0
6.5
6.0
5.5
5.0
4.5
4.0
3.5
3.0
2.5
2.0
1.5
1.0
ppm
5.34
1.00
1.11
1.06
1.59
1.74
3.81

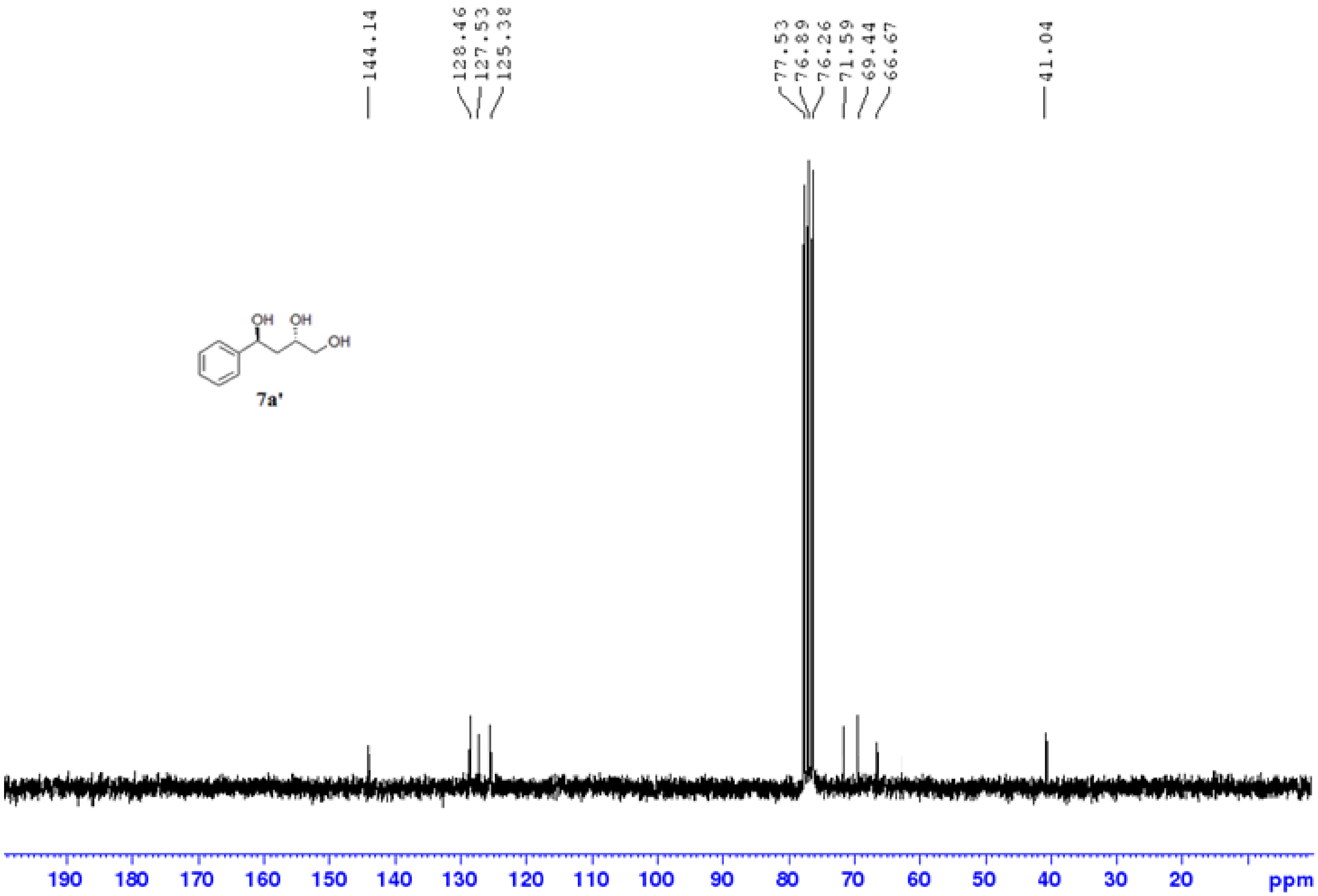
144.14
128.46
127.53
125.38
77.53
76.89
76.26
71.59
69.44
66.67
41.04
OH
OH
OH
7a'
190
180
170
160
150
140
130
120
110
100
90
80
70
60
50
40
30
20
ppm

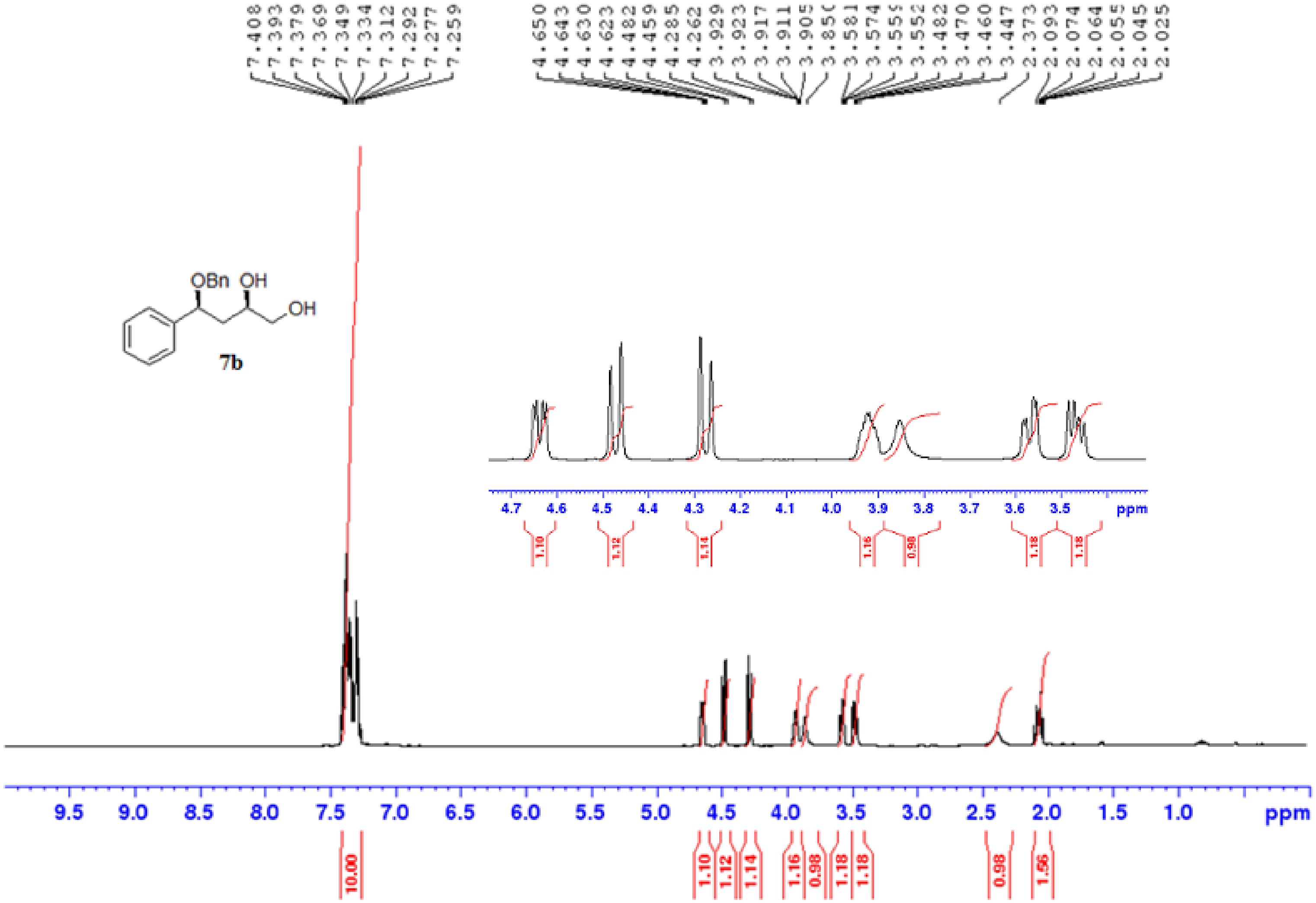

OBn OH
OH
7b
ppm

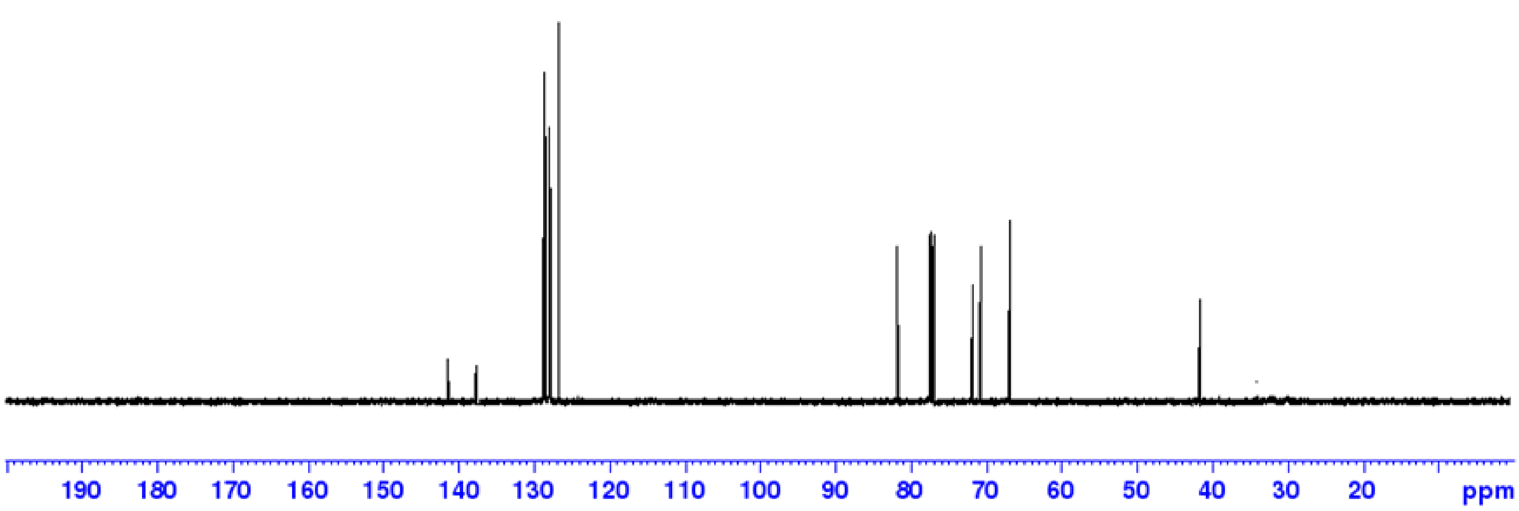
141.196
137.522
128.685
128.535
128.066
127.952
127.897
126.591
81.487
77.253
76.998
76.744
71.590
70.534
66.596
41.394
190
180
170
160
150
140
130
120
110
100
90
80
70
60
50
40
30
20
ppm

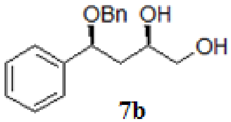
OBn
OH
OH

7b

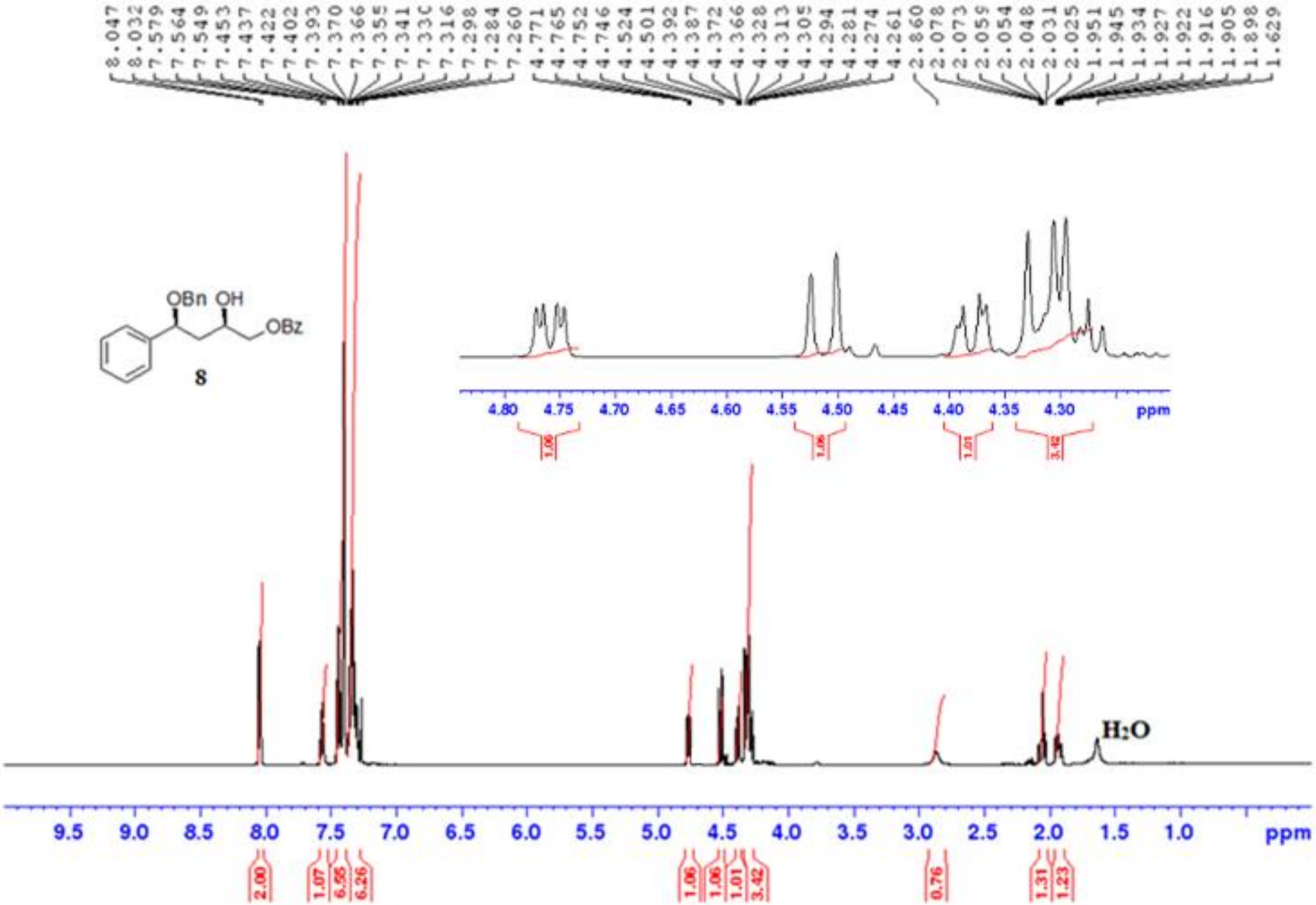
OBn OH
OBz
8
H₂O
ppm

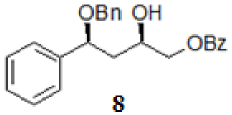
OBn OH
OBz

8

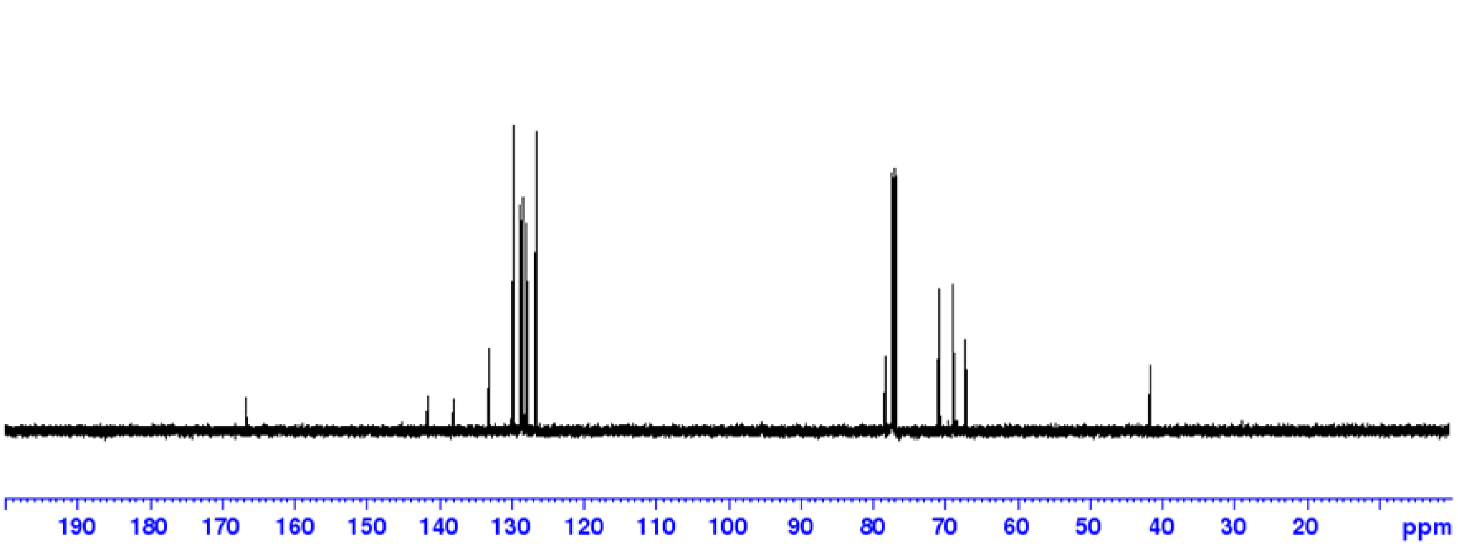
166.625
141.569
137.968
133.06
129.663
128.75
128.673
128.53
128.474
128.362
128.32
127.962
127.88
127.847
127.795
126.676
126.540
78.114
77.252
76.997
76.743
70.794
68.75
67.052
41.571
190 180 170 160 150 140 130 120 110 100 90 80 70 60 50 40 30 20 ppm

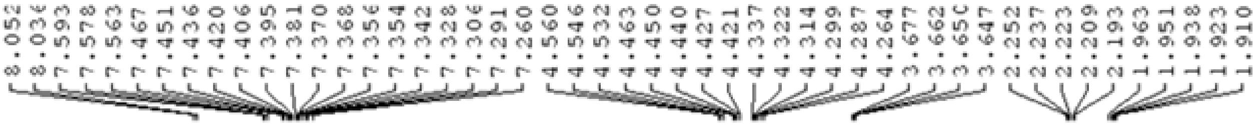
8.052
8.036
7.593
7.578
7.563
7.467
7.451
7.436
7.420
7.406
7.395
7.381
7.370
7.368
7.356
7.354
7.342
7.328
7.306
7.291
7.260
4.560
4.546
4.532
4.463
4.450
4.440
4.427
4.421
4.337
4.322
4.314
4.299
4.287
4.264
3.677
3.662
3.650
3.647
2.252
2.237
2.223
2.209
2.193
1.963
1.951
1.938
1.923
1.910

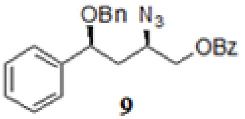
OBn
N3
OBz

9

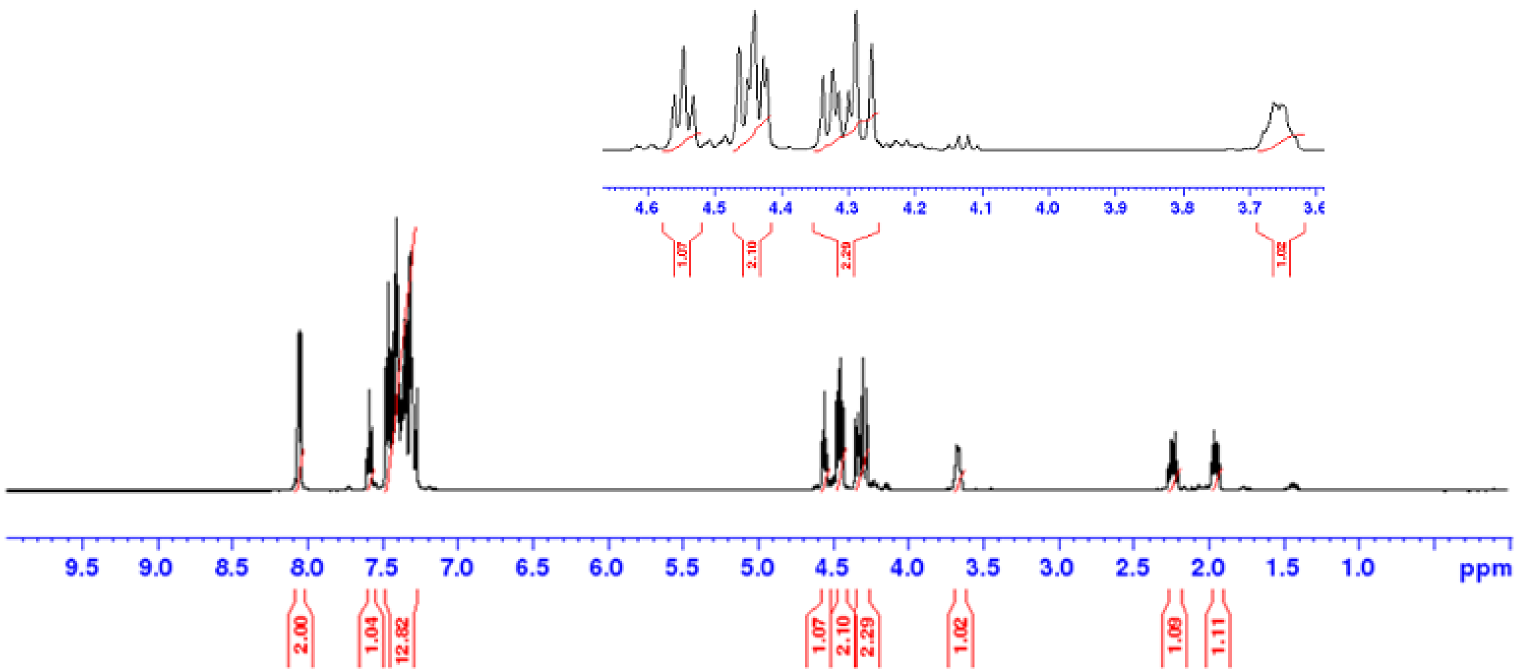
4.6 4.5 4.4 4.3 4.2 4.1 4.0 3.9 3.8 3.7 3.6
1.07 2.10 2.29 1.02
9.5 9.0 8.5 8.0 7.5 7.0 6.5 6.0 5.5 5.0 4.5 4.0 3.5 3.0 2.5 2.0 1.5 1.0 ppm
2.00 1.04 12.82 1.07 2.10 2.29 1.02 1.09 1.11

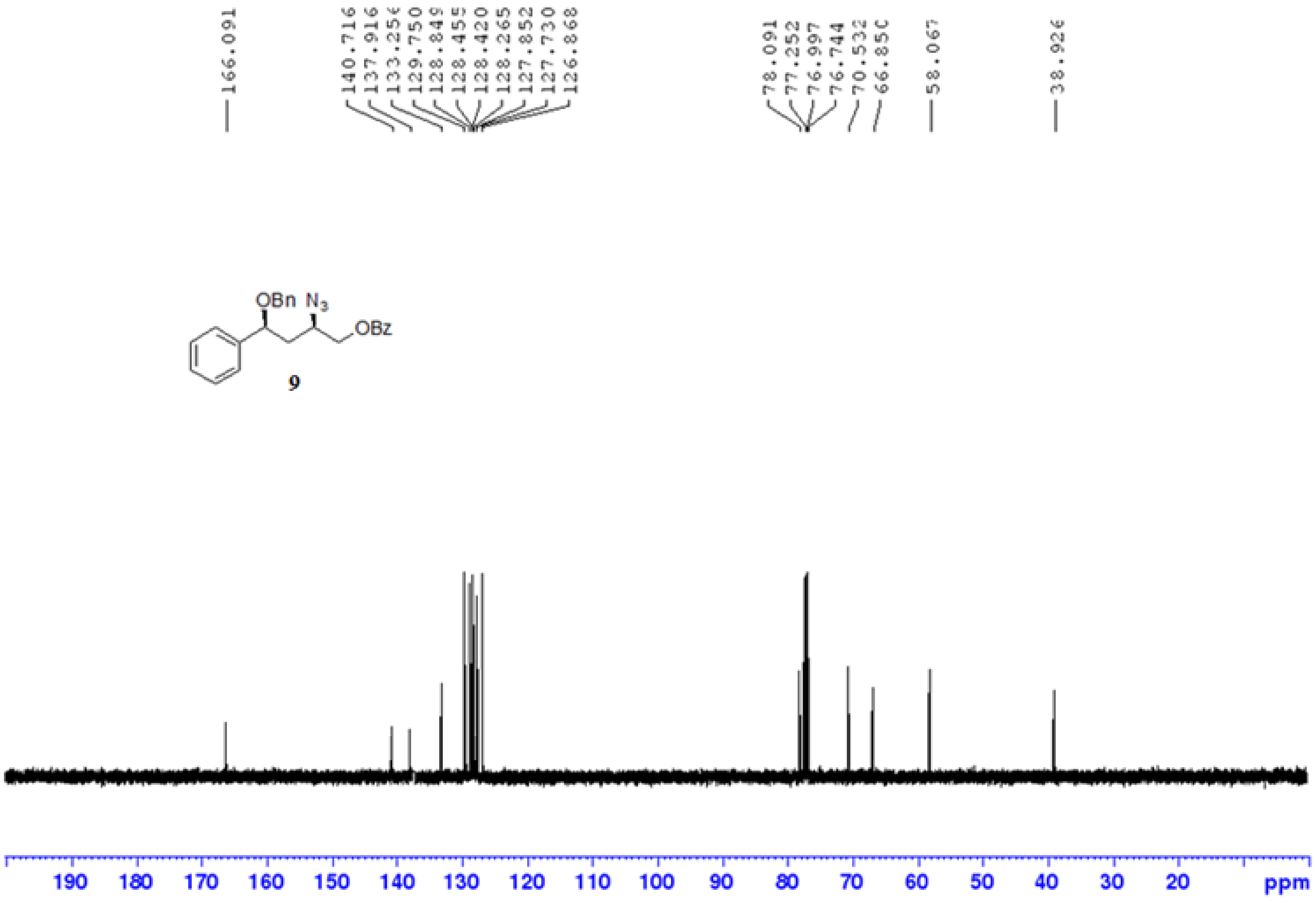

166.091
140.716
137.916
129.750
128.420
128.265
127.852
127.730
126.868
78.091
77.252
76.997
76.744
190 180 170 160 150 140 130 120 110 100 90 80 70 60 50 40 30 20 ppm
OBn
N_3
OBz

9

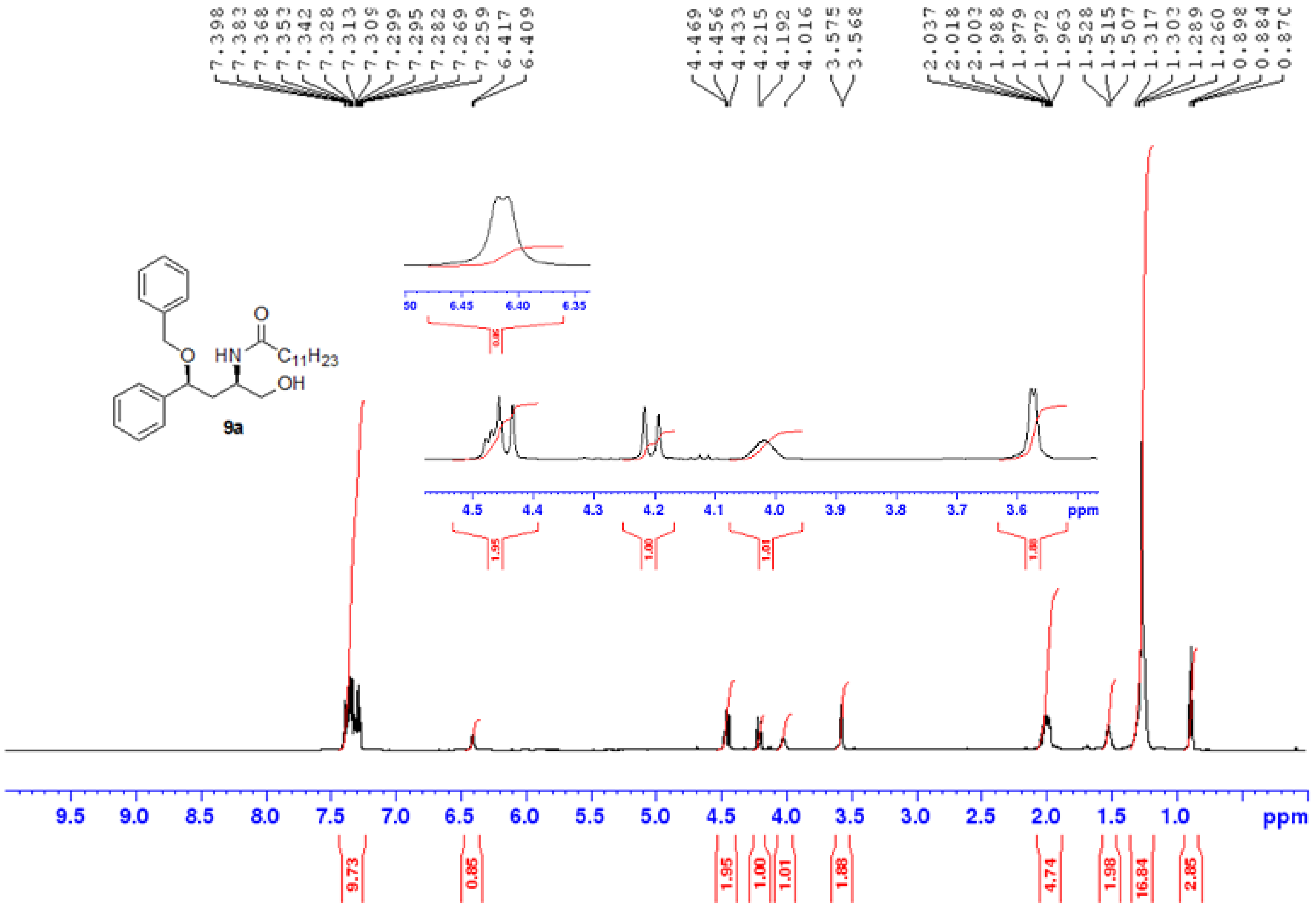

9a
ppm

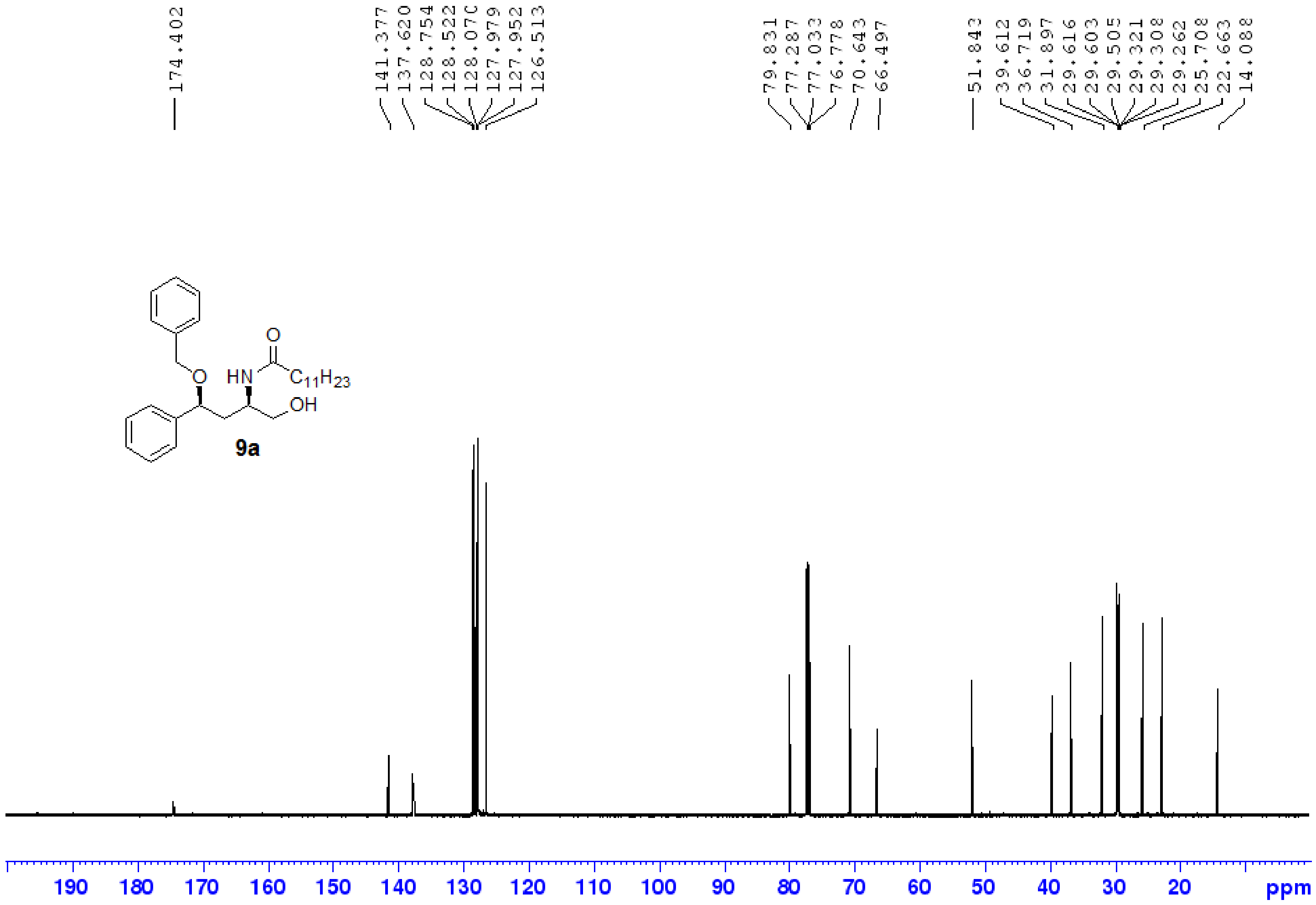
174.402
141.377
137.620
128.754
128.522
128.070
127.979
127.952
126.513
79.831
77.287
77.033
76.778
70.643
66.497
51.843
39.612
36.719
31.897
29.616
29.603
29.505
29.321
29.308
29.262
25.708
22.663
14.088
O
HN
C11H23
O
OH
9a
190 180 170 160 150 140 130 120 110 100 90 80 70 60 50 40 30 20 ppm

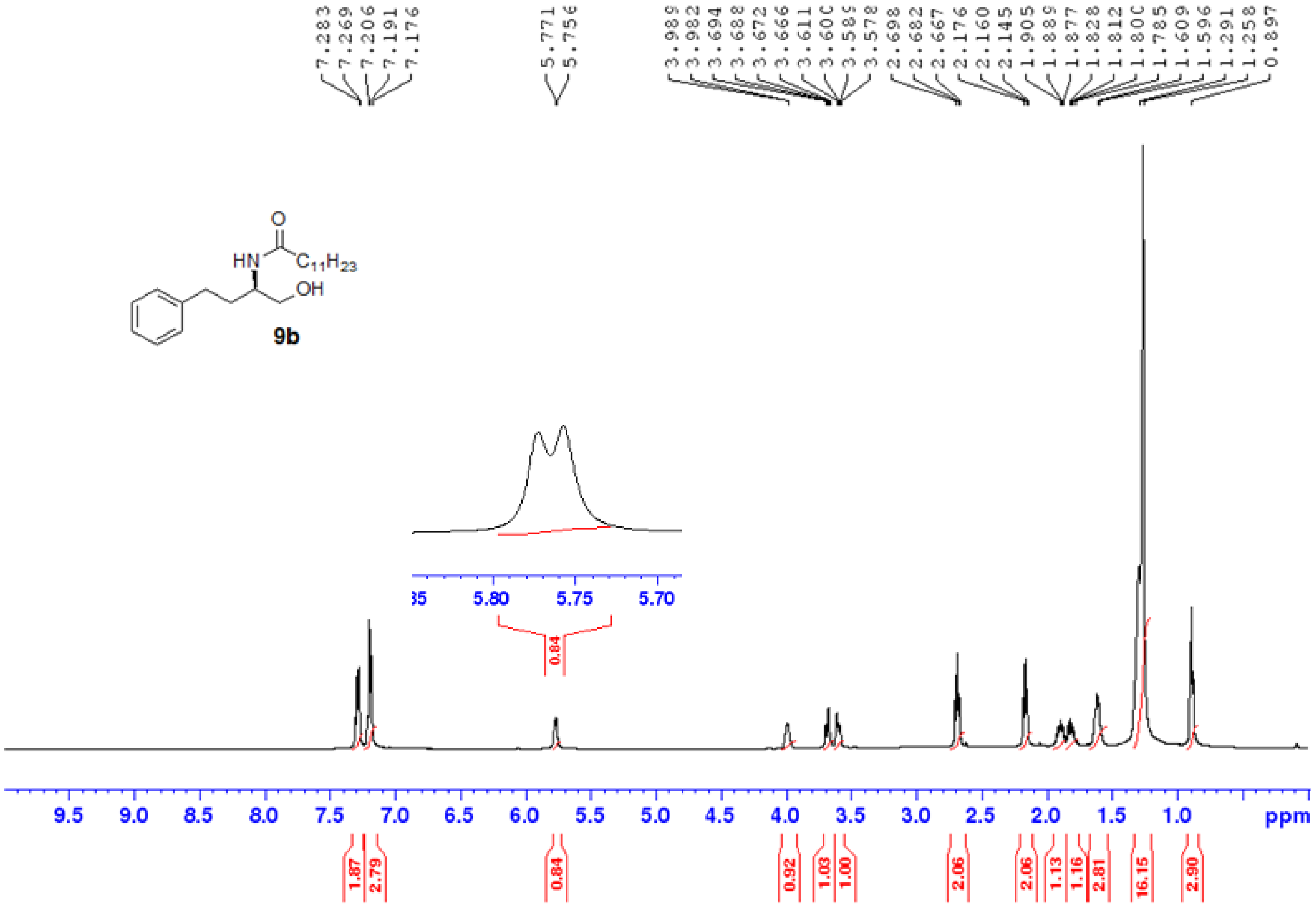
7.283
7.269
7.206
7.191
7.176
5.771
5.756
3.989
3.982
3.694
3.688
3.672
3.666
3.611
3.600
3.589
3.578
2.698
2.682
2.667
2.176
2.160
2.145
1.905
1.889
1.877
1.828
1.812
1.800
1.785
1.609
1.596
1.291
1.258
0.897
O
HN
$C_{11}H_{23}$
OH
9b
5.80
5.75
5.70
0.84
9.5
9.0
8.5
8.0
7.5
7.0
6.5
6.0
5.5
5.0
4.5
4.0
3.5
3.0
2.5
2.0
1.5
1.0
ppm
1.87
2.79
0.84
0.92
1.03
1.00
2.06
2.06
1.13
1.16
2.81
16.15
2.90

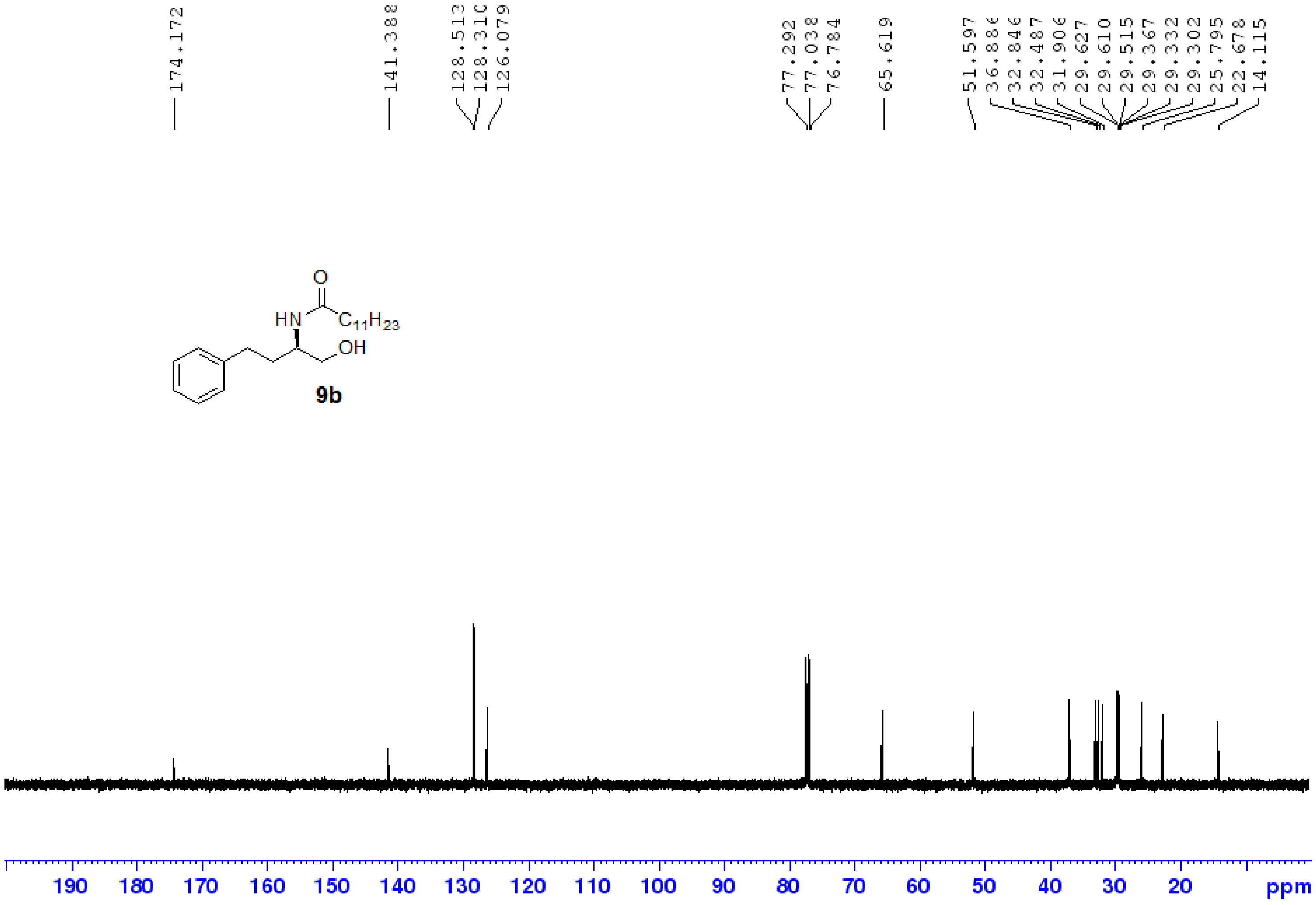
174.172
141.388
128.513
128.310
126.079
77.292
77.038
76.784
65.619
51.597
36.886
32.846
32.487
31.906
29.627
29.610
29.515
29.367
29.332
29.302
25.795
22.678
14.115
O
HN
$C_{11}H_{23}$
OH
9b
190 180 170 160 150 140 130 120 110 100 90 80 70 60 50 40 30 20 ppm

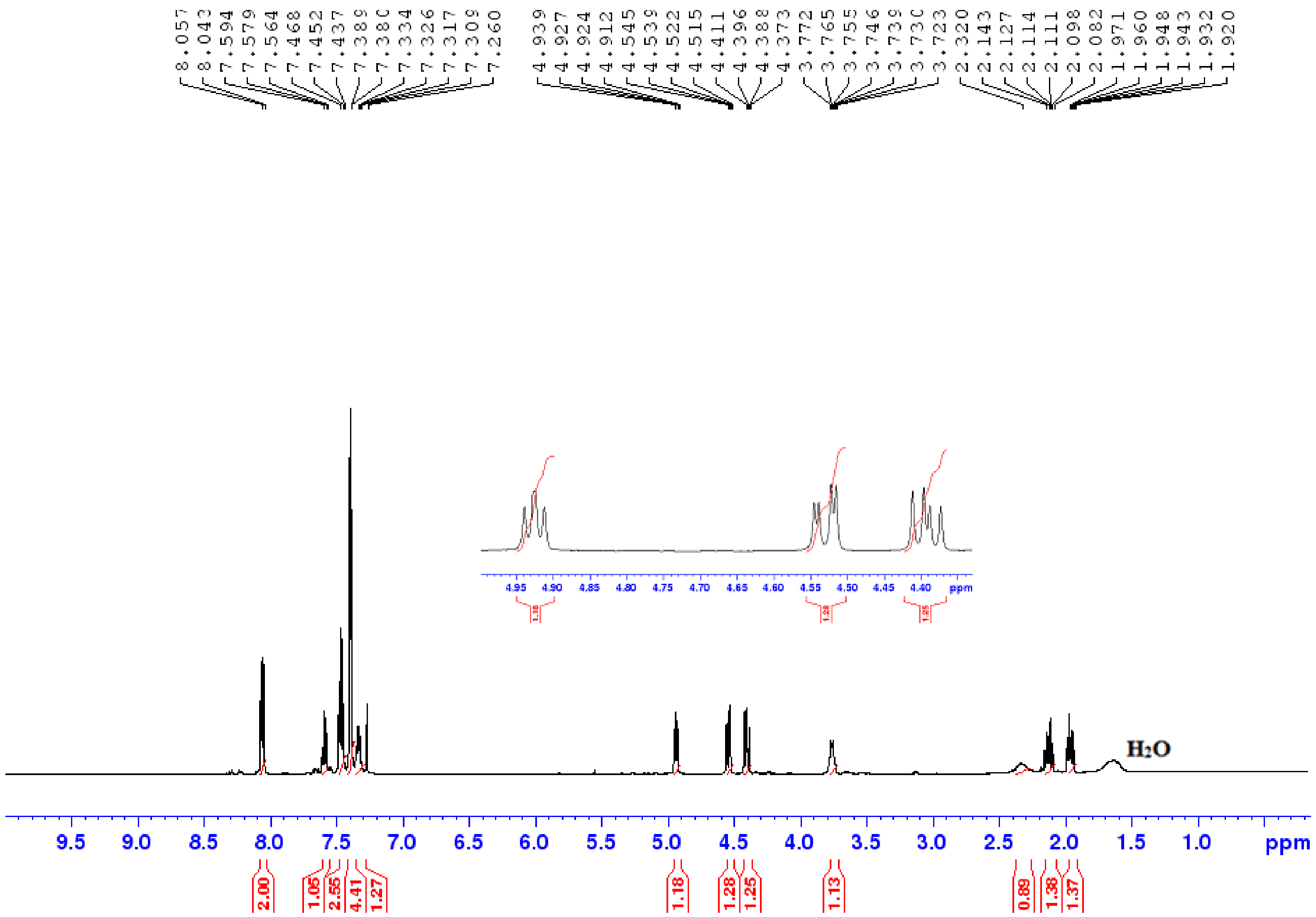

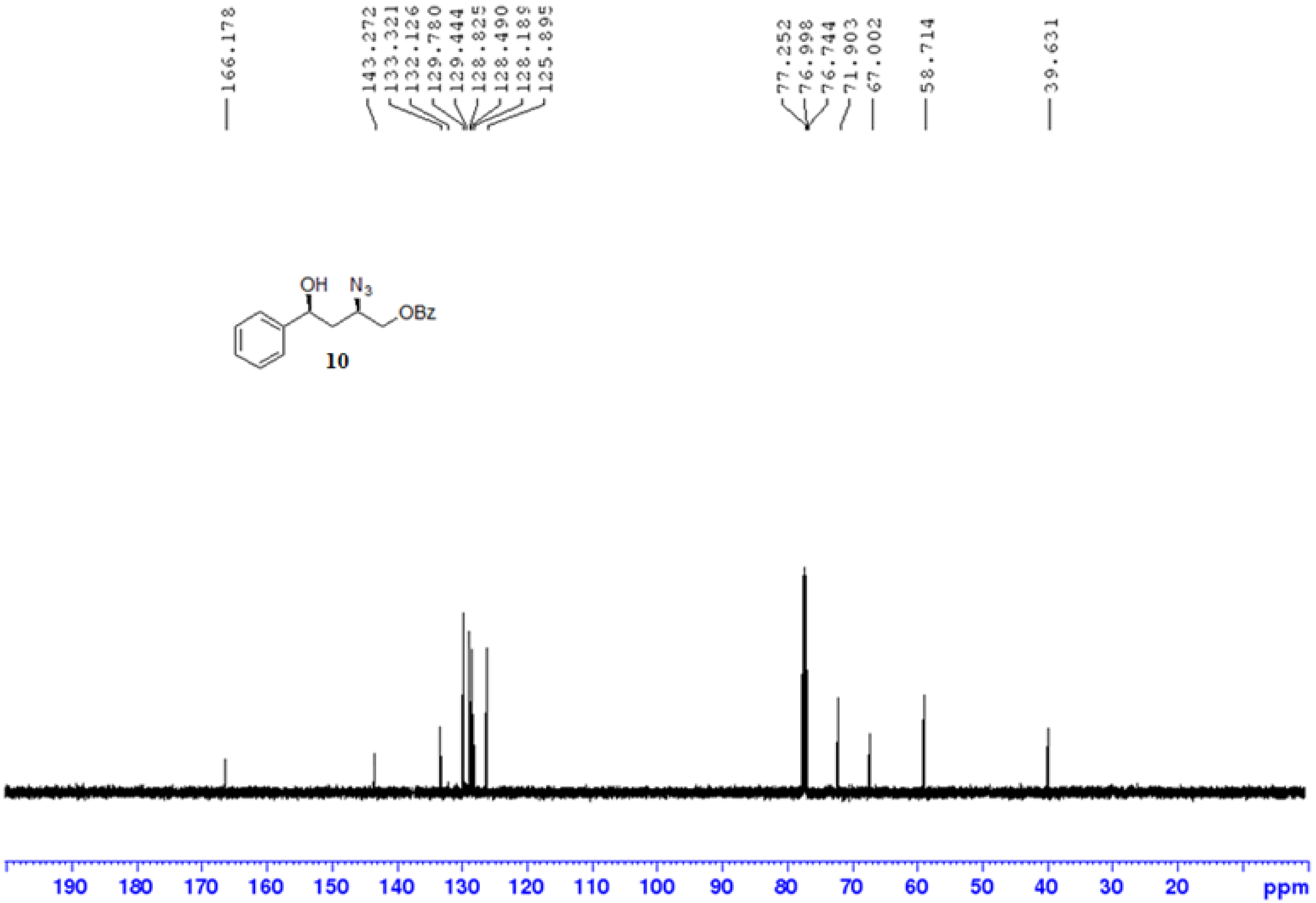
166.178
143.272
133.321
132.126
129.780
129.444
128.825
128.490
128.189
125.895
77.252
76.998
76.744
71.903
67.002
58.714
39.631
OH
N3
OBz
10
190
180
170
160
150
140
130
120
110
100
90
80
70
60
50
40
30
20
ppm

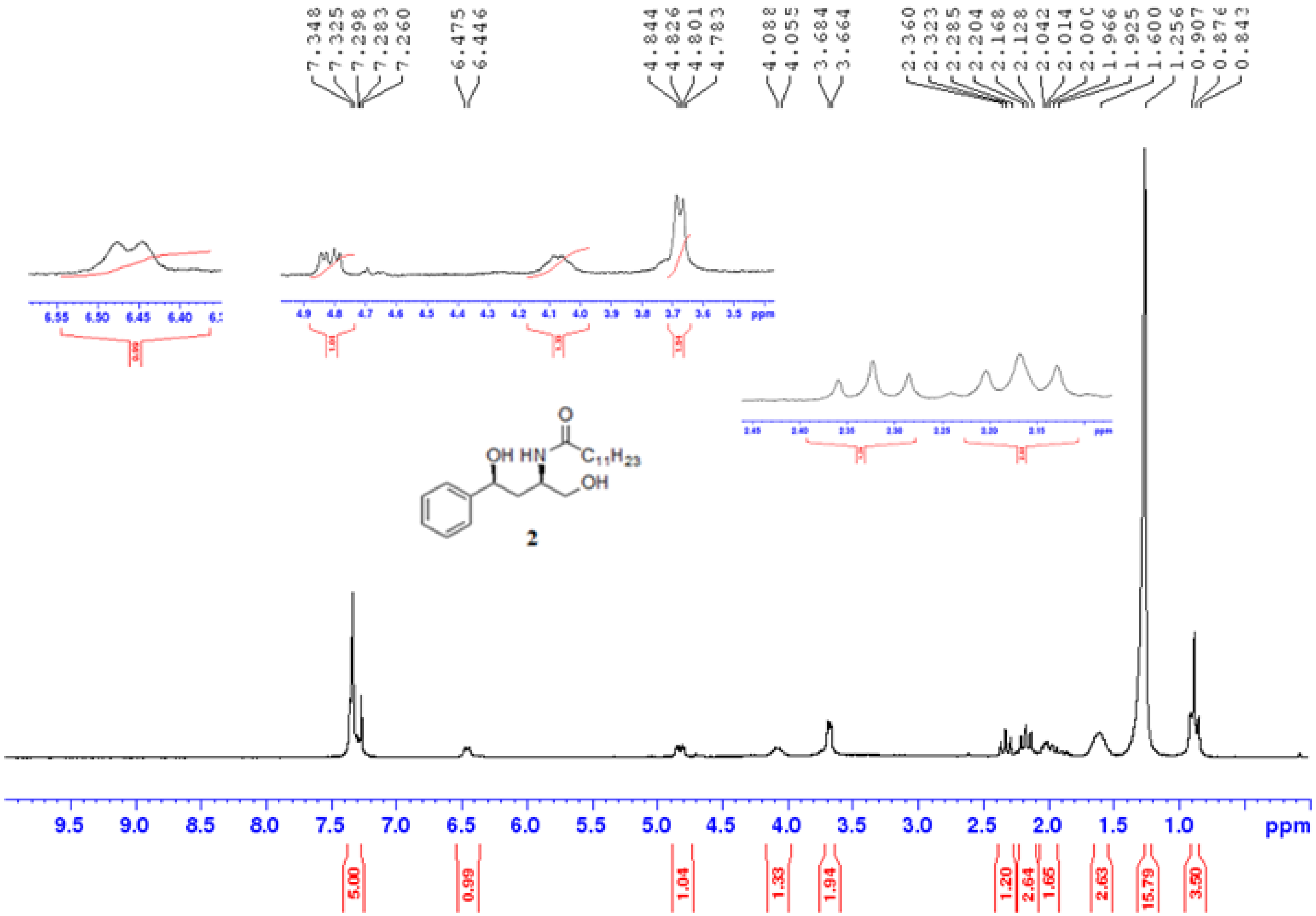

7.348
7.325
7.298
7.283
7.260
6.475
6.446
4.844
4.826
4.801
4.783
4.088
4.055
3.684
3.664
2.360
2.323
2.285
2.204
2.168
2.128
2.042
2.014
2.000
1.966
1.925
1.600
1.256
0.907
0.876
OH HN
O
$C_{11}H_{23}$
OH
2
9.5
9.0
8.5
8.0
7.5
7.0
6.5
6.0
5.5
5.0
4.5
4.0
3.5
3.0
2.5
2.0
1.5
1.0
ppm
5.00
0.99
1.04
1.33
1.94
1.20
2.64
1.65
2.63
15.79
3.50

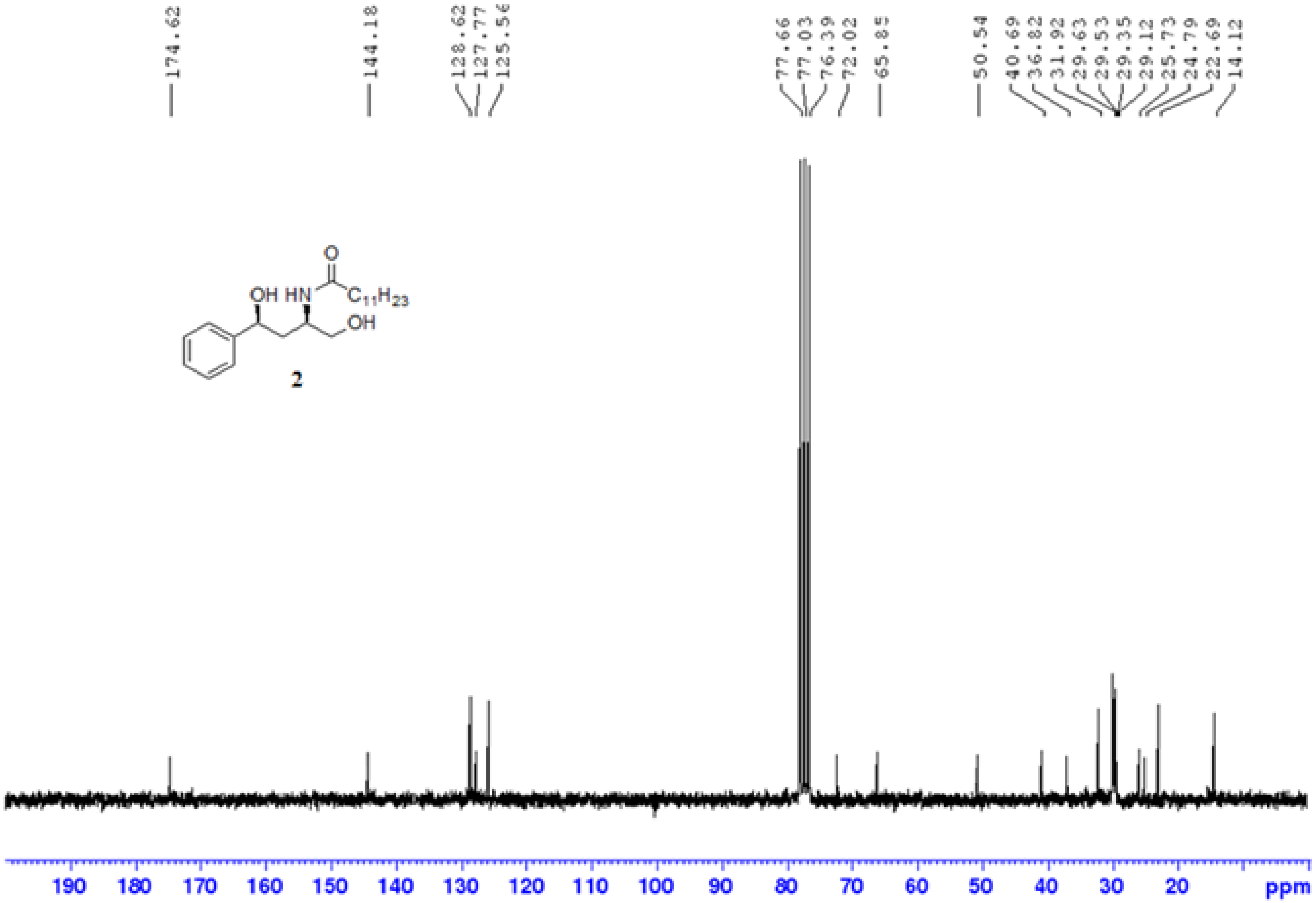
174.62
144.18
128.62
127.77
125.56
77.66
77.03
76.39
72.02
65.85
50.54
40.69
36.82
31.92
29.63
29.53
29.35
29.12
25.73
24.79
22.69
14.12
OH HN
O
$C_{11}H_{23}$
OH
2
190 180 170 160 150 140 130 120 110 100 90 80 70 60 50 40 30 20 ppm
